# Knowledge-based prediction of DNA hydration using hydrated dinucleotides as building blocks

**DOI:** 10.1107/S2059798322006234

**Published:** 2022-07-21

**Authors:** Lada Biedermannová, Jiří Černý, Michal Malý, Michaela Nekardová, Bohdan Schneider

**Affiliations:** a Institute of Biotechnology of the Czech Academy of Sciences, BIOCEV, Průmyslová 595, 252 50 Vestec, Czech Republic

**Keywords:** DNA hydration, water, dinucleotide fragments, knowledge-based prediction, *WatNA*

## Abstract

Water plays an important role in stabilizing the structure of DNA and mediating its interactions. Here, the hydration of DNA was analyzed in terms of dinucleotide fragments from an ensemble of 2727 nonredundant DNA chains containing 41 853 dinucleotides and 316 265 associated first-shell water molecules. The dinucleotides were classified into categories based on their 16 sequences and the previously determined structural classes known as nucleotide conformers (NtCs). The construction of hydrated dinucleotide building blocks allowed dinucleotide hydration to be calculated as the probability of water density distributions. Peaks in the water densities, known as hydration sites (HSs), uncovered the interplay between base and sugar-phosphate hydration in the context of sequence and structure. To demonstrate the predictive power of hydrated DNA building blocks, they were then used to predict hydration in an independent set of crystal and NMR structures. In ten tested crystal structures, the positions of predicted HSs and experimental waters were in good agreement (more than 40% were within 0.5 Å) and correctly reproduced the known features of DNA hydration, for example the ‘spine of hydration’ in B-DNA. Therefore, it is proposed that hydrated building blocks can be used to predict DNA hydration in structures solved by NMR and cryo-EM, thus providing a guide to the interpretation of experimental data and computer models. The data for the hydrated building blocks and the predictions are available for browsing and visualization at the website https://watlas.datmos.org/watna/.

## Abstract 2

Database-derived water probability densities around structurally and sequentially distinct DNA dinucleotide fragments reproduce the known hydration motifs, which thus can be used as building blocks to predict DNA hydration.

## 1. Introduction

Life evolved in an aqueous environment and water is the environment in which biomolecules perform their functions. Water not only acts as a solvent, but its molecules influence the structure, dynamics and interactions of biomolecules. An interdependence between the functions of biomolecules and their hydration waters is now well recognized (Chaplin, 2006 ▸; Raschke, 2006 ▸; Ball, 2008 ▸; Biedermannová & Schneider, 2016 ▸; Laage *et al.*, 2017 ▸; Persson *et al.*, 2018 ▸; Zsidó & Hetényi, 2021 ▸; Yamamoto *et al.*, 2021 ▸). Water molecules are known to influence folding and recognition between biomolecules (Levy & Onuchic, 2006 ▸; Hummer, 2010 ▸), and they mediate DNA–peptide recognition (Khesbak *et al.*, 2011 ▸) and interactions between DNA and minor-groove binders (Nguyen *et al.*, 2009 ▸; Wei *et al.*, 2013 ▸; Neidle, 2021 ▸). Release of partially ordered water from the hydrated layer upon the binding of interacting partners is a source of entropic force (Leikin *et al.*, 1993 ▸; Dunitz, 1994 ▸). Water molecules often mediate sequence-specific recognition by bridging the protein–DNA interfaces (Jayaram & Jain, 2004 ▸; Schneider, Černý *et al.*, 2014 ▸), and hydration of the DNA target is an important element in its recognition by the cognate protein (Shakked *et al.*, 1994 ▸).

The high affinity of DNA for water has been observed since the first fiber diffraction studies, notably in the seminal work of Franklin (Franklin & Gosling, 1953 ▸) and in experiments using various biophysical techniques (Wang, 1955 ▸; Tunis & Hearst, 1968*a* ▸,*b* ▸). In the following decades, it was demonstrated that there are differences between the properties of the solvent and counterions on the surface of nucleic acids and in the bulk (Texter, 1979 ▸; McDermott *et al.*, 2017 ▸; Chalikian *et al.*, 1994 ▸), and that the water content is an important factor in structural transitions between different DNA forms, as reviewed by Fuller *et al.* (2004 ▸). In addition, water molecules on the biomolecular surface and at the interface between two biomolecules have different dynamic properties (Schneider, Gelly *et al.*, 2014 ▸), but the exact physical mechanism by which the dynamics of a biomolecule and water are coupled is not fully understood (Heyden, 2014 ▸; Conti Nibali *et al.*, 2014 ▸). The interface water is known to participate in folding of the three-dimensional structure of nucleic acids (Sorin *et al.*, 2005 ▸) and to stabilize their native structure (Auffinger & Hashem, 2007*b* ▸) by creating well structured and interlinked water networks in double helices (Drew & Dickerson, 1981 ▸) and quadruplexes (Li *et al.*, 2021 ▸). Quite aptly, water was described as an ‘integral part of nucleic acids’ by Westhof (1988 ▸).

There is no universal definition of what actually constitutes the hydration layer specifically hydrating a biomolecule. The thickness of the layer is a matter of debate and depends on the technique used to probe the system. Estimates stretch from layers of one to several waters, and the thickness from 4 Å to greater than 20 Å (Ebbinghaus *et al.*, 2007 ▸). Fiber diffraction revealed localized sites of preferred hydration in the major groove of A-DNA (Langan *et al.*, 1992 ▸). Localized, slow-motion water molecules that were first reported in the B-DNA minor groove as ‘the spine of hydration’ (Drew & Dickerson, 1981 ▸) have been confirmed by several other methods, most notably NMR spectroscopy (Liepinsh *et al.*, 1992 ▸) and more recently nonlinear vibrational spectroscopy (McDermott *et al.*, 2017 ▸) and time-resolved fluorescence Stokes shift (Sardana *et al.*, 2019 ▸).

The most detailed data on the interface between waters and biomolecules are provided by X-ray and neutron crystallo­graphy. These methods provide detailed spatial and dynamic information about the one- or two-molecule-thick shell of water directly interacting with biomolecular surfaces. The very first DNA crystal structures revealed many well ordered interconnected waters in the minor grooves of Z-form (Wang *et al.*, 1979 ▸) and B-form (Drew & Dickerson, 1981 ▸) DNA and between phosphate groups in A-form duplexes (Saenger *et al.*, 1986 ▸), the direct comparison of B- and A-form hydration for high-resolution structures (Egli *et al.*, 1998 ▸) and water molecules forming extended hydrogen-bonded networks completely encapsulating the DNA–drug complex (Shieh *et al.*, 1980 ▸). An increase in the number of DNA-containing structures determined by X-ray crystallography in the early 1990s allowed the determination of structural motifs around small DNA building blocks such as bases (Schneider & Berman, 1995 ▸) and phosphates (Schneider *et al.*, 1998 ▸), but not for a nucleotide or a larger fragment. DNA bases bind many well ordered waters, while fewer structurally ordered waters are observed around the heavily hydrated but highly dynamic phosphate groups (Gessner *et al.*, 1994 ▸; Eisenstein & Shakked, 1995 ▸). An overview of the hydration shell observed by cryo-crystallo­graphy has been published (Nakasako, 2004 ▸), and structural patterns of nucleic acid solvation have subsequently been studied in the context of DNA structure and conformation by Auffinger & Hashem (2007*a* ▸).

These and other studies have shown that DNA hydration is sequence- and structure-dependent. The growing number of solved DNA structures and the corresponding increase in structural and sequence diversity available at the beginning of the 21st century provided an opportunity to analyze larger fragments than bases or phosphates in the generic A–B–Z duplex environment. The amount, quality and diversity of available structures allow the structural classification of dinucleotide fragments, which are generally considered to be the minimal structurally sensible fragment (Richardson *et al.*, 2008 ▸). DNA and RNA dinucleotide conformers are now comprehensively defined as NtC classes (Schneider *et al.*, 2018 ▸; Černý, Božíková, Svoboda *et al.*, 2020 ▸). One of the 96 geometrically defined NtC classes is assigned by an automated tool as a web service at https://dnatco.datmos.org (Černý *et al.*, 2016 ▸; Černý, Božíková, Malý *et al.*, 2020 ▸).

The NtC classes are structurally more relevant building blocks than bases and phosphates, which, even when analyzed in the context of the B, A or Z duplex architecture, had a limited power to explain the complex interplay between conformation and sequence. Due to the classification into the NtC classes and the manyfold increase in available crystal structures, analysis of DNA hydration is now possible as a function of the NtC-defined conformation and the 16 possible dinucleotide sequences.

In this work, we analyze a sequentially nonredundant set of DNA crystal structures to determine ‘hydrated building blocks’ for all sufficiently populated NtC/sequence combinations and analyze the hydration patterns in those that contain sufficient water molecules. Our detailed analysis concentrates on the most important and frequently observed DNA conformations, the BI and BII forms, and on the canonical A form; the other conformers are discussed wherever possible and appropriate. To test the applicability and predictive power of the derived data, we compared the crystal hydration of selected DNA oligonucleotides with the hydration patterns predicted by our NtC-based hydrated building blocks. We also predicted the water structure around a few structures solved by NMR. In analogy with our previously conducted amino-acid hydration analysis (Biedermannova & Schneider, 2015 ▸), we make the results, including both the hydrated building blocks and the hydration predictions, readily available for browsing and visualization in the form of the *WatNA* web application available at https://watlas.datmos.org/watna.

## 2. Methods

### 2.1. Selection of DNA structures

We selected a sequentially nonredundant set of DNA structures solved by X-ray crystallography at the highest possible crystallographic resolution. On 7 September 2020 we used the ‘advanced search’ and ‘create custom report’ features at https://www.rcsb.org/ (Berman *et al.*, 2002 ▸) and downloaded two sets of data: for all uncomplexed (sometimes called naked) DNA structures and for all protein–DNA complexes. The data were saved in JSON format and included information on PDB ID, chain, resolution, sequence, sequence length and cluster ID (an identifier of a cluster of proteins with a specified level of sequence identity). In-house Python scripts were used to extract the relevant information from the JSON files for structures containing at least six-nucleotide DNA strands. The structures were grouped based on their DNA sequences. Structures with sequences shorter than 24 nucleotides were allowed to differ at two positions, while longer sequences were allowed to differ at 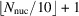 positions, where *N*_nuc_ is the number of nucleotides in the sequence. The results were written into a CSV table that contained additional information about the protein homology in the case of protein–DNA complexes.

The number of water molecules per nucleotide was calculated for the crystallographically unique DNA chains using a Python script for *UCSF Chimera* (Pettersen *et al.*, 2004 ▸); we only counted water molecules closer than 3.4 Å to any DNA atom, including symmetry-related atoms. The results were concatenated with the data in the CSV table. A Python script was used to search the CSV table for sequentially non­redundant DNA structures. In cases where there were several structures with the same or a similar sequence (a sequential cluster), the structure with the highest resolution was selected. If there were several structures with a similar resolution (±0.1 Å), the structure with the highest number of observed water molecules per nucleotide was selected. Thus, a set of sequentially nonredundant DNA chains (from both uncomplexed DNA and protein–DNA complexes) was obtained.

### 2.2. Extraction of hydrated dinucleotides

In the next step, we extracted dinucleotide fragments from the selected structures. The dinucleotides were classified into 96 NtC conformational classes; unassigned dinucleotides are formally placed into the 97th (NANT) class and are not further analyzed. The classification protocol has been described in detail previously (Schneider *et al.*, 2018 ▸; Černý, Božíková, Svoboda *et al.*, 2020 ▸; Černý, Božíková, Malý *et al.*, 2020 ▸). The combination of the 96 NtC classes and the 16 possible dinucleotide sequences resulted in 1536 NtC/sequence combinations. For each combination, we selected a reference dinucleotide structure using the classification at the https://dnatco.datmos.org website; we searched all DNA-containing PDB records, which currently comprise 4304 structures. The reference dinucleotide was selected as the dinucleotide with the lowest Cartesian r.m.s.d. with respect to the NtC class representative, choosing the dinucleotide with the highest confal score (Schneider *et al.*, 2018 ▸) within the r.m.s.d. group, if necessary.

**Figure 1:**
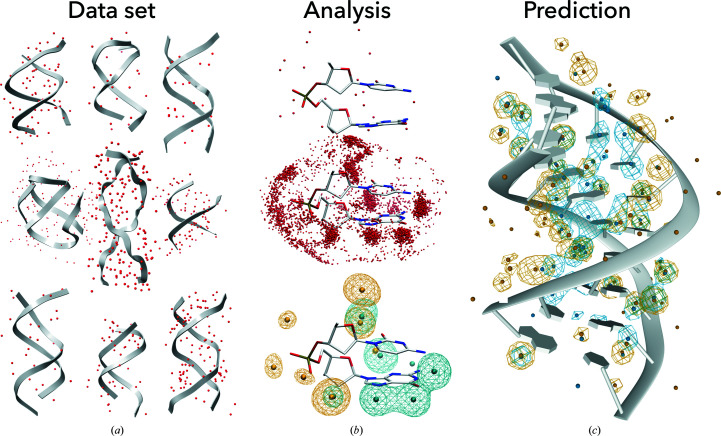
The procedure for determining DNA hydrated building blocks and their application for hydration prediction. (*a*) Selected structures from the analyzed data set of crystal structures from the PDB, containing 2727 nonredundant DNA chains (gray cartoon) together with the reported crystal water molecules (red balls). (*b*) Top: example of an extracted dinucleotide with water molecules closer than *d*_assoc_ = 4.0 Å. Middle: hydrated building block defined as the reference dinucleotide with the water distribution obtained by transferring all water molecules associated with the dinucleotides of the particular NtC/sequence combination. Bottom: 3D maps of water probability density calculated from the water distribution by Fourier averaging (Schneider *et al.*, 1998 ▸). Water probability density maps were calculated separately for waters closer than *d*_calc_ = 3.4 Å to the base (blue mesh) and to the sugar-phosphate atoms (yellow mesh) of the reference dinucleotide. Peaks in the hydration densities, shown as yellow and cyan spheres, are interpreted as hydration sites (HSs). (*c*) Water probability density maps calculated for PDB entry 6l75 (Jhan *et al.*, 2021 ▸) by overlapping the relevant hydrated building blocks over the crystal DNA structure and performing Fourier averaging.

In all analyzed structures the distances between nucleotide atoms and atoms labeled as water O atoms (further referred to as waters or water molecules) were calculated and waters at distances shorter than *d*_assoc_ = 4.0 Å were associated with the respective dinucleotides. Water positions were then transferred to the reference dinucleotide structure based on an alignment with the six dinucleotide atoms closest to the water in the original (nonreference) dinucleotide. This resulted in the hydrated building blocks: the reference di­nucleotides of a particular NtC/sequence combinati­on surrounded by an ensemble of waters found in all non­redundant crystal structures (Fig. 1 ▸).

### 2.3. Analysis of hydration patterns

Water molecules around the reference dinucleotides were transformed into probability densities using the previously published methodology of Fourier averaging (Schneider *et al.*, 1998 ▸) with the *CCP*4 program suite (Winn *et al.*, 2011 ▸). Fourier averaging represents a tool for obtaining the probability of water distributions around the analyzed fragments. Water distributions around the bases and around the sugar-phosphate backbone were calculated separately because they were significantly more ordered in the former than in the latter. Therefore, two sets of Fourier averaging calculations for water densities were performed: one for waters within 3.4 Å of any base atom of the respective reference dinucleotides and the other for waters within 3.4 Å of any atom of the sugar-phosphate backbone (Fig. 1 ▸ *d*). Hydration sites (HS), which are positions of high probability of water occurrence, were identified as peaks in the hydration density using *PEAKMAX* from the *CCP*4 suite. The occupancy of the HS was calculated as the number of water molecules within 1 Å of the HS divided by the number of dinucleotides for the given NtC/sequence combination. The resulting water probability densities and HS positions were visualized and figures were created in *UCSF Chimera* (Pettersen *et al.*, 2004 ▸), *ChimeraX* (Pettersen *et al.*, 2021 ▸), Mol* (Sehnal *et al.*, 2021 ▸) and *VMD* (Humphrey *et al.*, 1996 ▸).

### 2.4. DNA hydration prediction test

On 9 September 2021 we queried the PDB for high-resolution (<1.8 Å) X-ray structures of uncomplexed DNA and protein–DNA complexes with release dates later than 30 September 2020, *i.e.* after the acquisition of the set of structures for the hydration analysis. We assigned the NtC classes of these structures according to Černý, Božíková, Svoboda *et al.* (2020 ▸) and selected diverse structures with a low number of unassigned NtC dinucleotide conformers (NtC class NANT) and large numbers of crystallographically resolved water molecules.

In the selected structures, we overlaid the matching hydrated NtC blocks over dinucleotides in all structures using in-house *Bash* (https://www.gnu.org/software/bash/) and *VMD* (Humphrey *et al.*, 1996 ▸) scripts. The hydrated NtC blocks contained all of the extracted water molecules, and we scaled their occupancies to be inversely proportional to the number of dinucleotides for the particular NtC/sequence combination. In this way, the more populated hydrated NtC building blocks do not outweigh the less populated ones in the Fourier averaging procedure. The predictions were calculated separately for base- and backbone-related hydration, as described for the dinucleotide fragments in Section 2.3. The procedure resulted in a predicted water probability density map and predicted hydration sites for the whole structures. The predicted probabilities and positions of HSs were then compared with the positions of the crystallographically reported waters.

### 2.5. *WatNA*: an online atlas of DNA hydration

The *WatNA* web application was set up in analogy with our previous *WatAA* web application (https://watlas.datmos.org/wataa (Černý *et al.*, 2017 ▸), which analyzes water distributions around the 20 natural amino acids in the structural context of protein molecules. Details of the website construction can be found in the above reference; here, we concentrate only on features that differ between the *WatAA* and *WatNA* services.

*WatNA* is provided as an interactive web application that runs in a browser. The visualization component is based on a custom plugin for the Mol* viewer (Sehnal *et al.*, 2021 ▸). The use of standardized web technologies ensures the availability of *WatNA* across a wide spectrum of devices equipped with a current web browser. Technical infrastructure is provided by ELIXIR CZ. *WatNA* is available from https://watlas.datmos.org or directly at https://watlas.datmos.org/watna.

## 3. Results and discussion

### 3.1. The analyzed data set of DNA structures

The PDB (Berman *et al.*, 2002 ▸) search resulted in an initial set of 1100 X-ray structures of uncomplexed DNA and 4962 X-ray structures of protein–DNA complexes, with 637 of the former and 4641 of the latter being sequentially non­redundant; the criteria for selection among sequentially similar entries were high resolution and high water content, as described in Section 2. We then analyzed how the number of crystallographically resolved water molecules depends on the resolution of the structures. Not surprisingly, the water per nucleotide ratio shows a steep decline with decreasing resolution (Supplementary Fig. S1) and we decided to analyze only chains with a water per nucleotide ratio higher than 1.0 from structures of resolution equal to or better than 2.6 Å. The final set used for the analysis of hydration patterns included 497 chains of uncomplexed DNA and 2230 chains of DNA from protein–DNA complexes; a list of PDB codes used in the study is provided in Supplementary Table S1.

### 3.2. Hydration of the NtC dinucleotide classes

**Figure 2:**
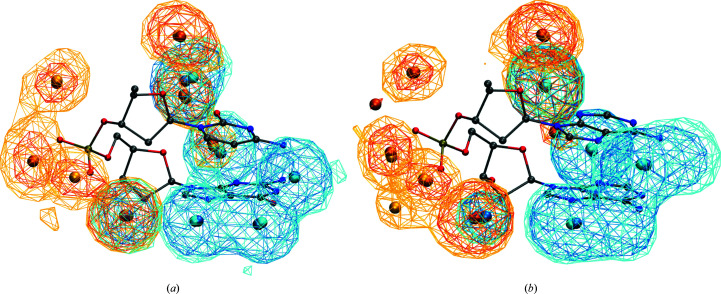
Water densities in uncomplexed DNA (light colors) and protein–DNA complexes (darker colors); the HSs are depicted as small balls. The water distributions associated with bases and backbone are shown in blue and yellow, respectively. Water densities around dinucleotides of (*a*) BB00/CG and (*b*) BB00/AA NtC/sequence combinations are shown.

Working with the final set of structures, each DNA chain was split into overlapping dinucleotide steps that included associated water molecules closer than 4.0 Å to any heavy atom of the dinucleotide. This procedure yielded 7524 and 40 877 hydrated dinucleotides for naked DNA structures and protein–DNA complexes, respectively. The former contained 69 212 and the latter contained 274 663 associated water molecules. We analyzed whether the hydration of uncomplexed and protein-associated DNA chains had different patterns. Of the NtC/sequence combinations that can be reliably analyzed for dinucleotides from less populated uncomplexed DNA structures, we investigated water densities and HS positions for BB00/CG and BB00/AA building blocks. Fig. 2 ▸ shows that the water distributions are very similar for dinucleotides from uncomplexed and complexed DNA; the corresponding HSs are also close to each other. Based on this analysis, we decided to pool the data for complexed and uncomplexed DNA and analyze them together.

Of the 48 401 dinucleotides in the pooled data set, 41 853 were assigned to one of the 96 NtC classes; the remaining 6548 could not be geometrically classified and cannot be further analyzed. The NtC-assigned dinucleotides contained 316 265 water molecules for which distributions were analyzed. The dinucleotides were sorted based on the 96 × 16 NtC/sequence combinations. The numbers of associated water molecules in NtC classes representing the A, BI and BII forms are listed in Table 1 ▸ and those for all NtC/sequence combinations are listed in Supplementary Table S2.

For each NtC/sequence combination, we calculated the water probability distribution by Fourier averaging, as described in Section 2. The large number of NtC/sequence combinations decreases the number of water molecules associated with individual combinations. While the NtC classes that are highly populated with DNA structures, such as BB00 or BB07, may have several thousand associated water molecules for a particular sequence, the less frequently occurring conformers such as NtC classes OPxx, ICxx and others have few if any water molecules associated with them, making it difficult to derive any meaningful information from their distributions. (For the numbers of NtC classes found in DNA structures across the database, see https://dnatco.datmos.org, ‘Table of conformers’ tab.)

After empirical visual inspection of many distribution maps, we determined a limit of about 800 associated water molecules for a given NtC/sequence combination as a reasonable threshold for reliable analysis of density distribution around both base and backbone atoms. According to this criterion, all 16 dinucleotide sequences can only be analyzed for NtC class BB00, corresponding to the canonical BI form of DNA (see Table 1 ▸, Supplementary Table S2 and the ‘BROWSE’ functionality of *WatNA*). The other two most important doublehelical DNA conformations, the BII and A forms (NtC classes BB07 and AA00, respectively), have significant sequence preferences (Schneider *et al.*, 2018 ▸) and their hydration can only be analyzed for about half of the dinucleotide sequences. For instance, the BII form (NtC class BB07) disfavors pyrimidine–pyrimidine and purine–pyrimidine steps, and these steps consequently have low numbers of associated waters. The sequence that is analyzable in most NtC conformers is GC followed by CG (Supplementary Table S1). In addition to the NtC conformers listed in Table 1 ▸, some of the NtC conformers that can reliably be analyzed (*i.e.* have more than 800 associated water molecules) are some sequences of B/A mixed conformers such as AB01 and BA05, B-form BB04, Z-DNA conformers ZZ1S and ZZS1, and the GG tetraplex conformer BBS1 in the GG sequence; they can be inspected using *WatNA*.

The ratio of water molecules per step does not show any obvious association with the NtC class or sequence (Table 1 ▸ and Supplementary Table S2). For example, in the BB00 conformational class the ratio varies between 6.4 and 9.6 and does not reflect any difference between purine and pyrimidine sequences. An apparently higher ratio of water molecules per step associated with A-form dinucleotides (namely NtC class AA00) and especially Z-form dinucleotides (NtC classes ZZS1 and ZZ1S, ratio of >10) is more likely to be a consequence of the higher crystallographic resolution of the associated crystal structures than to be an intrinsic property of these conformations. We therefore did not perform any statistical analysis of the significance of these differences.

### 3.3. Analysis of water-distribution maps

**Figure 3:**
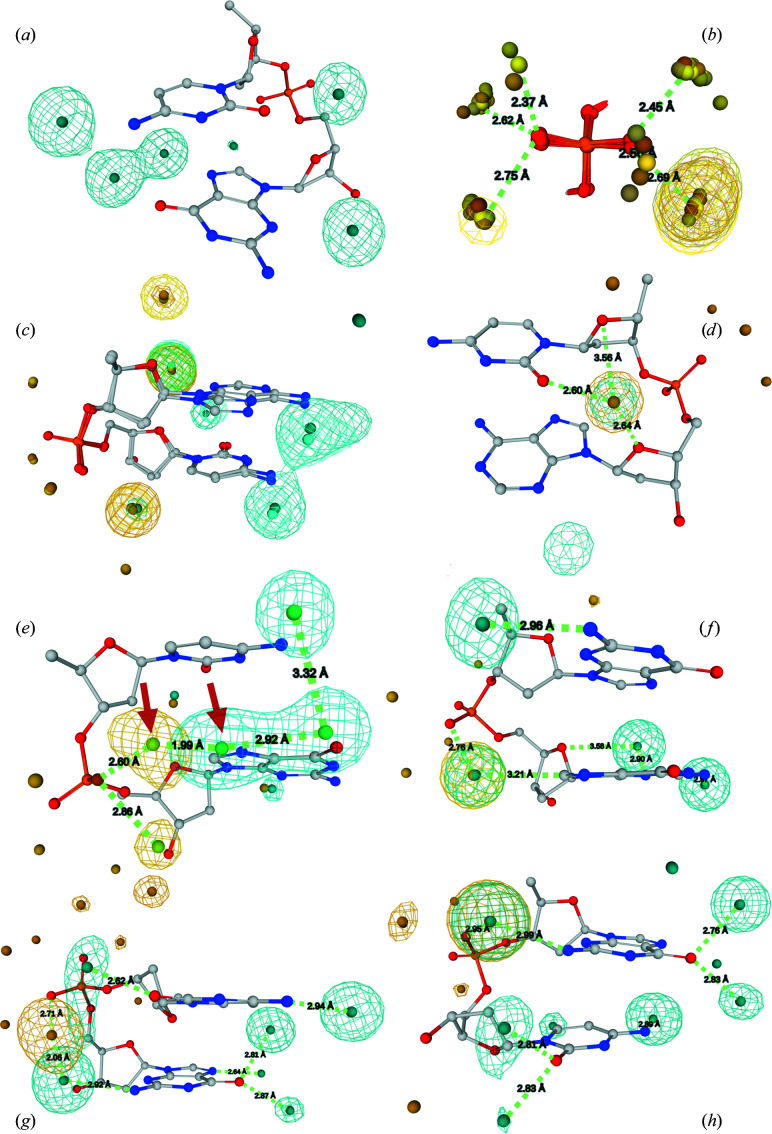
The hydration patterns of selected sequences of highly populated NtC classes. Water probability densities are displayed as a mesh and HSs as small balls. Base-related water density and HSs are displayed in blue and those for the backbone in yellow. These and other hydration patterns can be visualized using *WatNA*. (*a*) Typical hydration of bases in the BB00 class shown for the CG sequence. Pyrimidines have one HS in each groove; purines have two in the major groove and one in the minor groove. (*b*) HSs of the phosphate charged O atoms OP1 and OP2 in all 16 dinucleotide sequences of BB00, the NtC class best representing the BI form. Spheres in different shades of yellow represent HSs in different sequences; water densities are only displayed in matching colors for four of the sequences for clarity. The alignment shows three HSs for both OP1 and OP2 atoms. The most prominent HS in all sequences is the HS hydrating the OP2 atom. Waters constituting this HS are stabilized by van der Waals contact with the pyrimidine C6 or purine C8 base atom (base and sugar atoms are not shown for clarity). (*c*) Both NtC classes representing the BI form, BB00 and BB01, have similar structures and their hydration is also very similar. Data are for the AC sequence. (*d*) The BII form, NtC class BB07, has a unique HS in the minor groove. The HS is stabilized by contacts to N3(R)/O2(Y) of the first base and deoxyribose O4′ atoms of *both* sugar rings. The BII hydration is shown here for the CA sequence. (*e*) The hydration of the A form represented here by NtC class AA00 in the CG sequence. The most prominent difference relative to the hydration pattern of the BI form is the phosphate hydration, with two large water densities connected to the phosphate OP2 atom. One of these densities overlaps with the base hydration and water would fluctuate between the positions of the HSs highlighted by red arrows. (*f*) Hydration of the NtC class BBS1 occurring in the GG sequence, mostly in quadruplex structures. (*g*) The hydration pattern of the CG step of the Z form that is described by NtC class ZZ1S. (*h*) The hydration pattern of the GC step of the Z form, described by NtC class ZZS1. The hydration pattern of the left-handed DNA is very different from that in both right-handed forms: water densities are mostly located between the base planes rather than in the planes, and base and phosphate hydration merge in the minor rather than the major groove.

In the following paragraphs, we systematically examine the hydration patterns in the most prevalent DNA conformer, the BI form, and then relate these patterns to the patterns in the BII form and the canonical A form; other conformational classes such as those occurring in G-tetraplexes, Z-DNA or mixed B/A dinucleotides are examined as appropriate. As discussed above, only patterns based on a sufficient number of water-molecule positions can meaningfully be analyzed. We analyze water distributions in the form of pseudo-electron densities and HSs; illustrations of eight examples are shown in Fig. 3 ▸.

#### 3.3.1. Hydration of the BI form, NtC classes BB00 and BB01

*Hydration of the bases*. Hydration of the major groove has a simple pattern with HSs positioned approximately in the base planes (Fig. 3 ▸ *a*). Each of the two polar purine (R) atoms has a cloud of interacting waters with well defined HSs in guanine, whereas in adenine the water cloud interacting with the amino N6 atom may be weak. Each polar pyrimidine (Y) atom in the major groove, N4(C) and O4(T), is hydrated by a well defined water density with a localized HS. Both Y and R nucleotides have a strong focused hydration density between a hydrophobic C atom (C8 in R and C6 in Y) and the phosphate O atom OP2. In thymine, the HS is further sharpened by the methyl group; C8 of adenine apparently has a much lower affinity for water than C8 of guanine or C6 of both pyrimidines. The HS shared by the C8/C6 base and OP2 phosphate atoms and the accompanying strong water density is a unique feature of the BI form; it is observed in both NtC classes BB00 and BB01. In contrast, a similar HS does not exist in the BII and A forms.

The common feature of minor-groove hydration is a single HS for the hydrophilic atoms of Y and R; hydration of the guanine exocyclic N2 is weak. The cloud of waters hydrating O2(Y) and N3(R) also hydrates the deoxyribose O4′ atom of the following nucleotide in the sequence. In this way, the manner in which the base and deoxyribose are bridged by a water is sequence-dependent: the guanine N2(*i*) is bridged to O4′(*i* + 1) in GA, GC, GG and GT sequences, forming an HS distinct from the HS of N3. This HS adds extra stabilization to the water network by connecting the HSs of N3(*i*), N2(*i*) and N3/O2(*i* + 1). The minor-groove HSs in the other sequences are separated by more than 4 Å and are not connected.

*Phosphate and deoxyribose hydration*. Each of the partially charged phosphate O atoms, OP1 and OP2, potentially has three HSs forming the ‘cones of hydration’ that have been predicted theoretically (Pullman *et al.*, 1975 ▸; Langlet *et al.*, 1979 ▸), observed experimentally around phosphate charged O atoms in crystal structures (reviewed by Westhof, 1993 ▸) and confirmed in our previous hydration study (Schneider *et al.*, 1998 ▸). Not all three HSs are pronounced in all NtC/sequence combinations. The strongest HS linking OP2 to the major-groove base atom C8(R) or C6(Y) has already been discussed. The second HS of OP2 is weakly connected to the deoxyribose atoms from the preceding nucleotide, while the third, weakest HS has low density and is not detectable in some sequences (AA, GA); when present it connects to OP1 at van der Waals distances (∼3.6 Å). Water distributions around OP1, which points away from the DNA atoms, are more scattered and the corresponding HSs have lower densities; the highest density links OP1 to the O5′(*i*) atom. Water hydration probabilities around phosphates are only weakly sequence-dependent (Fig. 3 ▸ *b*).

Both NtC classes that define the BI form, BB00 and BB01, have similar conformations. Not surprisingly, their water densities also have similar distributions and the HSs have geometrically close positions (Fig. 3 ▸ *c*).

#### 3.3.2. Hydration of the BII form, NtC class BB07

*Hydration of the bases*. The hydration sites lie approximately in the base planes as in the case of the BI form. In purines, often just one HS hydrogen-bonded to N7 is pronounced and the water density connected to O6/N6 is lower. Both pyrimidines have one HS in each groove. The minor-groove HS and the corresponding density shared by the first base N3(R)/O2(Y) and O4′ of the following deoxyribose is present and strong in all sequences because the constituting water molecules are stabilized by interaction with O4′ of *both* deoxyriboses; the distance to O4′ of the first sugar is about 3.5 Å (Fig. 3 ▸ *d*). This hydration site is unique to the BII form.

*Phosphate and deoxyribose hydration*. In contrast to the BI form, the geometry of the OP2⋯W⋯C8(R)/C6(Y) bridge is suboptimal in the BII form. In consequence, the water densities between the phosphate OP2 and the hydrophobic base C8(R)/C6(Y) atoms are weaker than in the BI form. The cone of hydration with three HSs around each charged phosphate O atom is formed more clearly than in the BI form.

#### 3.3.3. Hydration of the canonical A form, NtC class AA00

A strong sequence preference of the A form for GC-rich sequences does not allow the analysis of all 16 dinucleotides (Table 1 ▸); the hydration of the CG dinucleotide is illustrated in Fig. 3 ▸(*e*).

*Hydration of the bases.* Hydration is still preferably located in the base planes, but a different stacking pattern of the bases can allow sharing of the water distribution between the stacked bases in some sequences, especially in RR sequences, but less in RY sequences and not in YR or YY sequences; the effect is best visible in the GG sequence. The minor-groove hydration is simple with one density distribution for each base. As in BB00, the hydration of the first base is shared with O4′ of the second deoxyribose, creating a high-density HS.

*Phosphate and deoxyribose hydration*. As in BB00/BB01, OP2 is hydrated by a strong and well ordered hydration cloud creating an HS in the plane of the second base, but it is further from C8(R)/C6(Y) than in the BI form (similarly to the BII form). Hydration of the A-form dinucleotides is sequence-dependent. In dinucleotides with NR (any purine) sequences, the OP2 HS is close (∼2 Å) to the HS hydrogen-bonded to N7 of the purine base. Therefore, the phosphate OP2 and the purine N7 atoms share one fluctuating water cloud, with water molecule(s) forming hydrogen bonds to one or the other DNA atom. With a pyrimidine as the second nucleotide in the sequence, the phosphate and base hydration are further apart and water molecules hydrogen-bonded to OP2 and to O4(C)/N4(T) can be present at the same time; the respective HSs are about 3 Å apart and can form a hydrogen bond.

The second OP2 HS is also strong and is located below the base plane; it bridges to the phosphate of the sequentially following nucleotide, bridging their charged O atoms (see Section 3.4.3). Both OP2 HSs can be occupied at the same time as their distance is >3.4 Å. While all three HSs of OP2 are well defined, the densities hydrating the other charged O atom, OP1, are weaker.

#### 3.3.4. Mixed B/A conformers, NtC BA05 and AB01

These two conformers mix structural features of the BI and A forms and their hydration patterns reflect features of these respective forms. The hydration patterns are modulated by the local arrangement of atoms. For instance, the region between the OP2 and major-groove atoms of the second base is more similar to the B form in AB01 and to the A form in BA05, and the hydration pattern reflects this similarity so that the hydration pattern reflects its immediate neighborhood (Schneider & Berman, 1995 ▸).

#### 3.3.5. Z-form hydration, NtC classes ZZ1S and ZZS1

Due to a strong sequence preference of this form for an alternating CG (or more generally YR) sequence, the data allow reliable hydration analysis of only two Z-DNA related NtC classes, namely ZZ1S and ZZS1 (Table 1 ▸). Not surprisingly, due to substantial differences between the left-handed Z form and both right-handed forms B and A, the hydration patterns of these DNA forms are also very different.

Hydration of the NtC class ZZ1S observed in the CG sequence (Fig. 3 ▸ *g*) is dominated by two strong and mutually close HSs, one linked to the OP2 atom and the other to the minor-groove base N2(G) atom. The distance between these two sites is ∼2.1 Å. Therefore, hydration is shared by these two atoms; a water molecule can hydrogen-bond to one or the other atom, fluctuating between the positions. Two hydration sites of the major-groove O6(G) atom are distinctly out of the guanine plane. One of these HSs is in van der Waals contacts with the C5 and C6 atoms of the first base (C).

The NtC class ZZS1 is observed in the GC sequence (Fig. 3 ▸ *h*). Interestingly, the prominent OP2 HS in this case does not form a bridge to the second base as in most other classes, but to the minor-groove N2 atom of the first base (G). As in ZZ1S, the two HSs of the major-groove O6(G) atom are both strongly out of the plane of the base; one of them is in van der Waals contact with N4(C).

#### 3.3.6. GG tetraplex conformer, NtC BBS1

This conformer class is predominantly observed in GG quadruplexes or alternatively in single-stranded DNA fragments with specific function. Similarly to the hydration of BB00/GG, the prominent hydration site of OP2 is in contact with the C8 atom of the second guanine. However, the rest of the hydration pattern is very different from that of the B form. The exocyclic N2 of the first guanine in this case points to the major groove and has one distinct HS, as do each of the N2 and N3 atoms of the second guanine (Fig. 3 ▸ *f*).

### 3.4. Hydrated dinucleotides as building blocks

#### 3.4.1. Overall accuracy of the predictions

We propose that the hydrated NtC blocks can be used to predict the hydration structure around DNA molecules. To test this hypothesis and evaluate the accuracy of the predictions, we queried the PDB for high-resolution (<1.8 Å) DNA structures which were not contained in the original set of structures used for the hydration analysis. Subsequently, we manually selected a test set of ten DNA structures with a large number of crystal water molecules and a low number of unassigned (NANT) NtC classes; three of the structures are of uncomplexed DNA and seven are protein–DNA complexes (Table 2 ▸). We overlaid the hydrated NtC blocks containing thousands of extracted water molecules over the selected DNA structures and used the Fourier averaging procedure to obtain hydration probability densities. As for the individual hydrated blocks, we then located HSs as peaks in the predicted hydration densities.

To evaluate the prediction accuracy, we analyzed the consensus between the predicted HSs and the crystallo­graphically observed water molecules by measuring the distance between each predicted HS and the nearest crystallographically observed water molecule. For this analysis, we considered water molecules in the asymmetric unit as well as symmetry-related water molecules generated using the *GENSYM* program from the *CCP*4 suite (Winn *et al.*, 2011 ▸). The predicted water densities, HSs and the experimentally observed waters can be viewed in the *WatNA* web application under the PREDICTIONS tab. All of these structures show a similar trend in that most of the predicted HSs are located within the 0.0–0.5 Å distance interval and a diminishing number of HSs are at longer distances. The fraction of HSs in the first two intervals, *i.e.* within 1.0 Å of a crystallographic water molecule, is greater than 40% for all structures except PDB entry 6lui, and for four out of the ten structures it is close to or above 60% (67.8%, 63.6%, 62.5% and 59.2% for PDB entries 7cby, 7jy2, 6x5d and 6l75, respectively).

**Figure 4:**
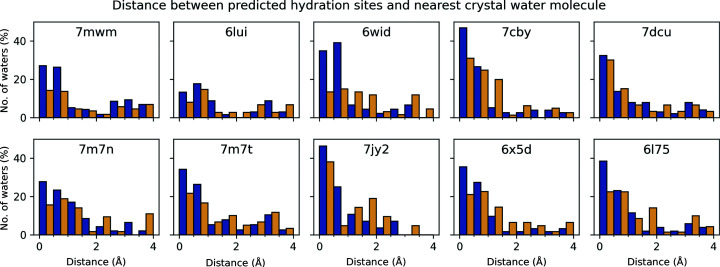
Histograms showing the percentages of predicted HSs located at a given distance interval from any of the crystallographically observed water molecules in the ten analyzed crystal structures listed in Table 2 ▸. Percentages for DNA base hydration are shown in blue and those for the sugar-phosphate backbone are in yellow. The graphs were created using *matplotlib* (Hunter, 2007 ▸).

The fractions are even higher for the HSs predicted solely for the DNA bases (73.9%, 73.4% and 71.4% for PDB entries 6wid, 7cby and 7jy2, respectively; Fig. 4 ▸). Not surprisingly, the prediction is much less accurate for the sugar-phosphate part of the DNA. However, for some of the structures the fraction of HSs within 1.0 Å of a crystallographic water molecule is still high enough to be useful (55.6%, 45.2%, 42.9% and 45.0% for PDB entries 7cby, 7dcu, 7jy2 and 6l75, respectively). Interestingly, for the joint X-ray/neutron high-resolution (1.0 Å) structure with PDB entry 7jy2, a large fraction of the sugar-phosphate HSs are located within the 0.0–0.5 Å distance interval. This shows that our prediction can be reliable even for the sugar-phosphate part of DNA and that the consensus between the predicted and observed water positions also depends on the quality of the crystal structure. Therefore, we propose that the Fourier averaging procedure using the hydrated NtC blocks is a viable method for prediction of the overall DNA hydration structure.

Next, we investigated how well the predicted hydration patterns reproduce the known features of DNA hydration, such as the ‘spine of hydration’ (Drew *et al.*, 1981 ▸; Kopka *et al.*, 1983 ▸), the ‘double string of hydration’ (Privé *et al.*, 1987 ▸), the ‘economy of hydration’ in the A form relative to the B form (Saenger *et al.*, 1986 ▸) and the features of Z-DNA hydration (Harper *et al.*, 1998 ▸).

#### 3.4.2. Spine of hydration in B-DNA

The spine of hydration is a feature of the B-DNA form and was first reported in the Drew–Dickerson dodecamer (Drew *et al.*, 1981 ▸; Kopka *et al.*, 1983 ▸). The spine of hydration is a zigzag string of water molecules in the narrow minor groove. It is formed by a row of first-shell water molecules that bridge the N2(R) or O2(Y) atoms of bases from opposing strands and by second-shell water molecules that in turn bridge the first-shell water molecules. Later, a feature called the double string (or ribbon) of hydration was reported in wider minor grooves of B-DNA, which are present in both AT and GC regions of the helix (Privé *et al.*, 1987 ▸).

**Figure 5:**
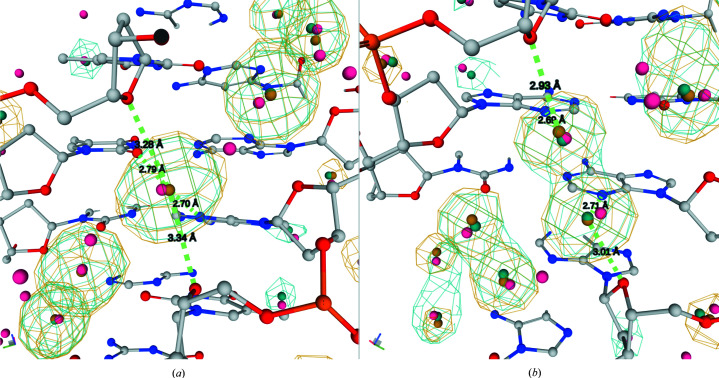
Prediction of the hydration structure in the minor groove of B-like DNA duplexes. (*a*) The predicted hydration reproduces the first-shell portion of the spine of hydration in the AT-rich region of PDB entry 7cby (Dai *et al.*, 2020 ▸) between the T12T13 step of chain *A* base-paired to the A4A5 step of chain *B*. (*b*) Double string of hydration reproduced in PDB entry 7cby (Dai *et al.*, 2020 ▸). Crystal water positions are shown as red spheres and predicted hydration sites for the bases and sugar-phosphate backbone are shown as cyan and yellow spheres, respectively; the corresponding hydration probability densities are shown as cyan and yellow mesh. Figures are clipped for clarity. The predicted hydration densities for PDB entry 7cby and nine others can be viewed interactively using the *WatNA* web application.

We observed the first-shell portion of the spine of hydration in the predicted hydration patterns in several of the B-DNA structures in the test set (see the PREDICTIONS tab of *WatNA*). The second-shell part of the spine could not be predicted due to the cutoff of 3.4 Å used for the Fourier averaging procedure. For example, the first-shell portion of the spine of hydration was predicted to occur between the A11–T14 stretch of chain *A* and the A4–G7 stretch of chain *B* in the structure with PDB code 7cby (Fig. 5 ▸ *a*). The predicted HSs lie in the center of the minor groove between the base planes, being stabilized by the polar atom [O2(Y) and N3(R)] of the second base in each step and the O4′ atom of the deoxyribose following the step. The predicted spherical densities and the HS positions correspond closely to the positions of crystallographically observed water molecules.

In regions of the double helix where the minor groove is wider, it can fit a double string of waters. Often the spine of hydration and the double string of hydration were observed within the same structure. For example, in the structure with PDB code 7cby, a double string of hydration was predicted between T8A9 of chain *A*, which are base-paired to T8A9 of chain *B* (Fig. 5 ▸ *b*). Here, two HSs lie in plane with the bases and are stabilized by their polar atoms [N3(R)/O2(Y)] and by the O4′ atoms of the following nucleotides. As in the case of the spine of hydration, in the case of the double string of hydration the predicted HSs are also in good agreement with the crystallographically observed water positions.

#### 3.4.3. ‘Economy of hydration’ in the A-DNA form

**Figure 6:**
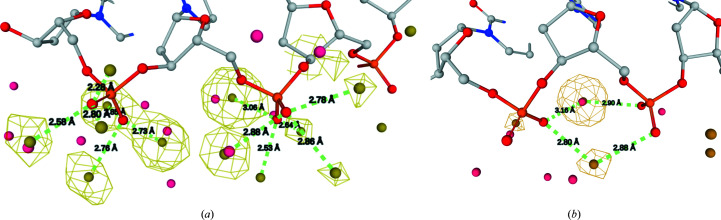
The predicted water probabilities and HSs correctly reproduce differences between phosphate hydration in the A and B forms. (*a*) Phosphate hydration in the A-DNA structure with PDB code 6l75 (Jhan *et al.*, 2021 ▸). The phosphates are bridged by water molecules in accordance with the ‘economy of hydration’ principle. (*b*) Phosphate groups hydrated separately in the B-DNA structure with PDB code 7m7n (Gregory *et al.*, 2021 ▸).

The predicted hydration patterns correctly reproduced an important difference between the BI, BII and A forms; namely, the difference in how two consecutive phosphate groups are hydrated (Fig. 6 ▸). The feature is known and has been termed the ‘economy of hydration’ (Saenger *et al.*, 1986 ▸). The shorter distances between phosphate O atoms in A-DNA lead to the possibility of forming water bridges between them. Thus, fewer water molecules are required to hydrate A-DNA compared with B-DNA, where the phosphates are hydrated individually. In the structure with PDB code 6l75, we observe HSs bridging OP1(*i*) and OP2(*i* + 1) atoms and also water molecules linking the OP2(*i*, *i* + 1) atoms of the phosphate groups in the major groove of A-DNA (Fig. 6 ▸ *a*), as described by neutron scattering studies (Langan *et al.*, 1992 ▸). In B-DNA structures, such as PDB entry 7m7n, the predicted hydration patterns formed a ‘cone of hydration’ for each of the OP1 and OP2 atoms, which were separate for the consecutive phosphate groups (Fig. 6 ▸ *b*).

#### 3.4.4. Hydration of the Z-DNA form

The predicted hydration of the Z-DNA structure with PDB code 7jy2 is characterized by a spine of water density in the minor groove, with HSs forming O2(C)⋯HS⋯O2(C) bridges joining cytosines across the strands (Supplementary Fig. S2). This feature had already been correctly predicted in the 1990s (Harper *et al.*, 1998 ▸) and was confirmed by the current results, where the predicted hydration sites are in good agreement with the water positions solved by neutron diffraction (PDB entry 7jy2; Harp *et al.*, 2021 ▸). Now, with hydration data for both the base and backbone, we observe a spherical water density HS forming a bridge between the N2(G) minor-groove atoms and the OP2 atom of the following phosphate. This new prediction is also in good agreement with the water positions in the crystal structure. Major-groove hydration is characterized by O6(G)⋯HS⋯O6(G) water bridges and N4(C)⋯HS⋯HS⋯N4(C) double water bridges between guanines and cytosines of the opposing strands, respectively (Supplementary Fig. S3).

#### 3.4.5. Water structure prediction in NMR-solved structures

We predicted the hydration structure in five DNA structures solved by NMR. The predictions can be inspected using the *WatNA* web application.

The NMR method is excellent for determining timescales for the hydration interactions but it does not provide accurate information about water locations. Among all 867 uncomplexed DNA structures solved by solution NMR (in the PDB as of 24 May 2022) only seven contained any water molecules. Of these seven structures, four of them (PDB entries 1a9g, 1a9h, 1a9i and 1a9j) contained only one water molecule per structure. PDB entries 1l0r, 1qch and 1llj contained hundreds (PDB entry 1llj) or even thousands (PDB entries 1l0r and 1qch) of water molecules modeled using molecular-dynamics simulations. Only a few of them were identified by NOE and ROE cross-peaks as having long residence times in the case of PDB entry 1qch. There were 141 structures of protein–DNA complexes, only four of which contained any water molecules. Again, there were either very few water molecules present (ten and one in PDB entries 1f4s and 1f5e) or these were modeled by molecular-dynamics simulation (PDB entries 1lcc and 1lcd). Modeling of the hydration structure in NMR-solved and modeled DNA structures can therefore be useful for a deeper understanding of their functioning.

### 3.5. *WatNA* web server

The data presented in this work are available for interactive inspection at the *WatNA* web application available at https://watlas.datmos.org/watna. *WatNA* is a part of the *watlas* server https://watlas.datmos.org, which currently provides two applications: the previously published *WatAA* application (https://watlas.datmos.org/wataa; Černý *et al.*, 2017 ▸) containing data on the hydration of amino-acid residues in proteins (Biedermannová & Schneider, 2015 ▸) and the *WatNA* application dedicated to the hydration of DNA dinucleotides presented here.

The navigation bar at the top of the *WatNA* webpage contains the following tabs: A-B-Z, BROWSE, PREDICTIONS, ABOUT, CONTACTS, HOW TO CITE, DOWNLOADS and WATLAS.

**Figure 7:**
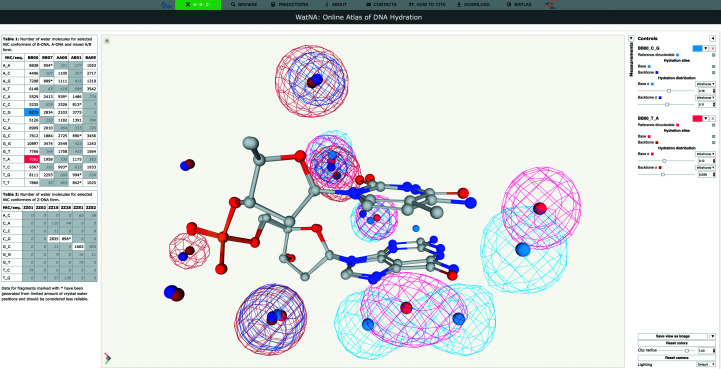
Screenshot of the A-B-Z tab of the *WatNA* application. The left column contains the interactive data table, the middle column contains the visualization panel with the Mol* viewer (Sehnal *et al.*, 2021 ▸) and the right column contains the Measurements panel (collapsed) and the Controls panel. Hydration of the BB00 CG and TA dinucleotides is visualized. The reference dinucleotide structures are shown in a ball-and-stick representation and HS positions are shown as colored spheres; the hydration probability distribution is contoured at a selected pseudo-occupancy level using a colored mesh.

The layout of the A-B-Z, BROWSE and PREDICTIONS tabs is divided into three sections: clickable data tables, a Mol* viewer (Sehnal *et al.*, 2021 ▸) and the Measurements and Controls panels. The A-B-Z tab (Fig. 7 ▸) presents data for the most populated NtC conformers of the A, B, mixed B/A and Z forms. The tables report numbers of water molecules observed in the NtC/sequence combinations. Clicking on a cell in the data table displays the corresponding dinucleotide, water densities and HSs in the viewer. Multiple NtC/sequence combinations can be displayed simultaneously for visual comparison; only data based on more than 800 water molecule positions are considered to be sufficiently reliable to be made available for viewing. The Controls panel allows the user to toggle display of the dinucleotide, HSs and density maps and to adjust the level of water densities. The colors of the displayed objects can be changed by clicking on the corresponding color box. The current state of the viewer can be saved as a PNG image by clicking on the ‘Save view as image’ button; more options are available after clicking on the arrow next to the button. The Measurements panel allows selected elements to be displayed to measure distances, angles and dihedrals between atoms and HSs.

The BROWSE tab is similar to the A-B-Z tab, but instead of displaying hydration patterns of a selected set of NtC/sequence combinations it allows the display of all combinations with more than 800 associated water molecules. The PREDICTIONS tab showcases the predicted hydration patterns calculated for the set of ten DNA-containing crystal structures listed in the caption to Fig. 4 ▸ and five NMR structures. The Controls panel in this tab enables the components of the original crystal structure (the DNA, protein and crystal water, and the predicted hydration sites and predicted hydration distribution) to be displayed/hidden to compare the predicted and crystal water positions.

The ABOUT tab presents a brief summary of the methods used in this work and of the possible application of the data. The HOW TO CITE tab contains information on how to cite the work and contacts for the authors, and the DOWNLOAD tab offers downloads of most of the data presented in the atlas. Additional data can be obtained from the authors by email or post as specified in the ABOUT tab. The WATLAS button on the navigation bar links to the overarching website https://watlas.datmos.org.

## 4. Conclusions

In the present work, we performed a comprehensive analysis of the hydration of DNA. We analyzed the hydration patterns around structurally and sequentially distinct dinucleotide fragments. The dinucleotides represent much larger and functionally more meaningful fragments than the isolated bases (Schneider & Berman, 1995 ▸) and phosphates (Schneider *et al.*, 1998 ▸) used in previous studies. Our analysis confirmed the findings of these previous studies, but this time we fully investigated the specific hydration features in the context of 16 dinucleotide sequences and conformations of the NtC classes and the interplay between base and backbone hydration.

The greater granularity of the analysis was enabled by a larger number of DNA crystal structures being available in the public archive and by the previously established classification of nucleic acid structures into dinucleotide conformational classes (NtCs; Schneider *et al.*, 2018 ▸; Černý, Božíková, Svoboda *et al.*, 2020 ▸).

We extracted water positions around dinucleotides in an ensemble of a nonredundant set of DNA structures containing 2727 DNA chains. The ensemble contained 41 853 dinucleotides that were classified into one of the 96 NtC structural classes. These dinucleotides have 316 265 associated water molecules. For each NtC/sequence combination, we calculated probabilities of the water distribution by the Fourier averaging procedure (Schneider & Berman, 1995 ▸) separately for base- and backbone-associated water molecules. The highest peaks in the water probability density maps represent the preferred sites for water positions called hydration sites (HSs; Fig. 1 ▸).

Many NtC/sequence combinations are infrequent or even absent in the DNA structures available in the PDB, so that the associated water densities cannot be calculated or reliably evaluated (Supplementary Table S2 and the BROWSE tab of *WatNA*). We estimated that analyses of dinucleotides with more than about 800 associated water molecules convey reliable information about the water density around the fragment. Significantly, water distributions can reliably be determined for most sequences of the most relevant DNA conformations, the BI, BII and A forms, represented by NtC classes BB00, BB07 and AA00, respectively. A few representative NtC/sequence combinations are depicted in Fig. 3 ▸; all can be inspected using *WatNA*.

To enable the easy visualization and inspection of all data presented in this paper, we created an online atlas of DNA hydration at https://watlas.datmos.org/watna. The atlas allows readers to access and visualize both the hydrated dinucleotide blocks (NtC/sequence combinations) and hydration predictions for whole DNA crystal structures.

We tested the predictive power of the hydrated dinucleotides by using them as building blocks for modeling DNA hydration in a set of ten DNA structures (Table 2 ▸). All of the structures show the similar trend that most of the predicted hydration sites are located within a 0.5 Å distance of the closest experimental water (Fig. 4 ▸). In addition, the predictions correctly reproduced known features of DNA hydration such as the first layer of the ‘spine of hydration’ in the B-DNA minor groove (Kopka *et al.*, 1983 ▸; Fig. 5 ▸), ‘economy of hydration’ in the A form relative to the B form (Saenger *et al.*, 1986 ▸; Fig. 6 ▸) and the intricacies of Z-DNA hydration (Harper *et al.*, 1998 ▸; Supplementary Figs. S2 and S3).

The good correlations between the experimental and predicted water positions give us confidence that the hydrated building blocks correctly reflect the interplay between the sequences, structures and hydration of DNA molecules. We predicted water structure around five NMR-solved structures to demonstrate that the building blocks can be used as a tool in modeling DNA structures and in deeper understanding of their structure–function relationships.

We propose that the Fourier averaging procedure using hydrated NtC blocks is a viable method for prediction of the overall DNA hydration structure. These predictions can serve as guidance in the interpretation of data from cryo-EM and NMR experiments and in the enhancement of theoretical models, as well as in the refinement of X-ray crystal structures. The predictions performed in this study can be viewed using the *WatNA* web application under the PREDICTIONS tab. In the future, we plan to develop the current approach into a fully automated prediction server for public use.

## 5. Data availability

The presented data are available from the *WatNA* web application at https://watlas.datmos.org/watna.

## Supplementary Material

Supplemtary Tables and Figures. DOI: 10.1107/S2059798322006234/cb5136sup1.pdf

## Figures and Tables

**Table 1:** Number of associated water molecules (‘number’) and average number of water molecules per dinucleotide (‘ratio’) for the given sequence/NtC combination Values are listed for the most frequent A-form conformer AA00, B-form conformer BB00 and the BII-form conformer BB07. Values for all 96 NtC conformer classes are given in Supplementary Table S2.

	AA00		BB00		BB07	
	Number	Ratio	Number	Ratio	Number	Ratio
AA	321	8.5	8838	7.1	1365	7.7
AT	618	8.8	6148	6.7	205	8.1
AC	1100	7.9	4496	6.6	254	5.9
AG	1111	8.6	7208	6.9	1372	8.4
TA	739	8.4	7082	7.4	1097	6.8
TT	463	6.2	7866	6.9	121	7.4
TC	993	8.7	6567	6.7	348	5.4
TG	693	10.2	8111	7.5	1615	9.0
CA	939	8.5	5529	6.9	1089	7.3
CT	1182	7.4	5126	6.2	333	6.2
CC	3326	7.4	5235	6.0	464	7.4
CG	2103	8.3	8275	8.0	2469	7.9
GA	464	6.3	8909	7.1	1700	7.2
GT	1758	8.6	7766	7.2	282	7.8
GC	2725	8.5	7612	7.4	936	9.1
GG	2549	8.1	10897	8.1	1936	9.3

**Table 2:** The set of ten DNA structures selected for testing hydration predictions

PDB code	Resolution (Å)	No. of HOH	DNA form		Reference
6lui	1.8	103	B-DNA	Protein–DNA	Stielow *et al.* (2021 ▸)
6l75	1.6	124	A-DNA	DNA	Jhan *et al.* (2021 ▸)
6wid	1.5	354	Mixed A/B	Protein–DNA	Kaminski *et al.* (2020 ▸)
6x5d	1.1	128	B-DNA	DNA	To be published†
7cby	1.6	351	B-DNA	Protein–DNA	Dai *et al.* (2020 ▸)
7dcu	1.7	438	B-DNA	Protein–DNA	Feng *et al.* (2021 ▸)
7jy2	1.5‡	201	Z-DNA	DNA	Harp *et al.* (2021 ▸)
7mwm	1.6	289	B-DNA	Protein–DNA	To be published§
7m7n	1.3	524	B-DNA	Protein–DNA	Gregory *et al.* (2021 ▸)
7m7t	1.5	584	B-DNA	Protein–DNA	Gregory *et al.* (2021 ▸)

† A structure of the Dickerson–Drew dodecamer with a 2′-MeSe-ara-T modification.

‡ Joint X-ray/neutron diffraction structure; the positions of heavy water, D_2_O, are reported.

§ Crystal structure of MBD2 with DNA.
