# Supplementary material for: Knowledge-based prediction of DNA hydration using hydrated dinucleotides as building blocks
Source: Acta Crystallogr D Struct Biol. 2022 Jul 21;78(Pt 8):1032–45. doi: 10.1107/S2059798322006234 (PMC9344474; doi:10.1107/S2059798322006234)
Supplement: Supplementary file 1 [file d-78-01032-sup1.pdf]

STRUCTURAL BIOLOGY

Volume 78 (2022)

Supporting information for article:

Knowledge-based prediction of DNA hydration using hydrated dinucleotides as building blocks

Lada Biedermannová, Jiří Černý, Michal Malý, Michaela Nekardová and Bohdan Schneider

Table S1. List of PDB codes of structures used in the study.

Uncomplexed DNA, listed as pdbID_chain:

3p4j_A, 1i0t_A, 1d8g_A, 4ocb_A, 4hig_A, 5jzq_A, 1dpl_A, 1ick_A, 4hif_A, 3ggk_A, 1enn_A, 4mj9_A, 4x1a_A, 1sk5_A, 1em0_A, 293d_A, 2elg_A, 352d_A, 4r15_A, 2dcg_A, 4e1u_A, 6niz_A, 1dpn_A, 3omj_A, 1i0m_A, 4qio_A, 1i0k_A, 1kgk_A, 3ggi_A, 3u89_A, 4x18_A, 4iii_A, 6gld_B, 5jeu_A, 292d_A, 1dcg_A, 1dj6_A, 131d_A, 5xjz_A, 1qyl_A, 1d48_A, 6aqt_A, 336d_A, 3wbo_A, 5zat_A, 3i5e_A, 1en3_A, 1en8_A, 1ene_A, 1en9_A, 5jev_A, 436d_A, 4i9v_A, 5gsk_A, 5wsq_A, 6mc2_A, 1fd5_A, 1d11_A, 1n1o_A, 460d_A, 2fih_A, 4l25_A, 1mf5_A, 367d_A, 284d_A, 2o1i_A, 1m69_A, 1fq2_A, 1jgr_A, 3opi_A, 3nz7_A, 440d_A, 427d_A, 1vro_A, 2f8w_A, 1d39_A, 2obz_A, 1r68_A, 3ft6_A, 6m2p_A, 4mkw_A, 2dyw_A, 431d_A, 4jd8_A, 244d_A, 145d_B, 1o0k_A, 3fl6_A, 3fl6_B, 3nyp_A, 3i5l_A, 1d35_A, 4fp6_A, 1d8x_A, 3ltr_A, 4f4n_A, 3u2n_A, 3ifi_A, 4agz_A, 6cq3_A, 454d_A, 4ah0_A, 2fii_A, 3u08_A, 3u05_A, 3ey3_A, 4c64_A, 3u0u_A, 4c63_A, 4c5x_A, 476d_A, 4u8b_A, 4u8c_A, 5lit_A, 145d_D, 362d_A, 4i1g_A, 145d_A, 4r49_A, 1m77_A, 6dy5_A, 3iki_A, 1z7i_A, 4r4d_A, 232d_A, 1vty_A, 1d61_A, 1dn5_A, 4fs5_A, 4fs6_A, 1omk_A, 6aqw_A, 6aqv_A, 1dc0_A, 1d40_A, 1d76_A, 1d41_A, 3ltu_A, 3dnb_A, 3hg8_A, 1p20_A, 2d34_A, 1zf1_A, 210d_A, 355d_A, 455d_A, 1ei4_A, 4l26_A, 463d_A, 4gju_A, 5w20_A, 4ah1_A, 403d_A, 2b3e_A, 6jje_A, 1zf8_A, 1zf9_A, 5et2_A, 5ix7_A, 245d_A, 1ims_A, 151d_A, 152d_A, 2plo_A, 224d_A, 1d54_A, 6jkn_A, 6ip3_A, 1qyk_A, 4qkk_A, 1ue2_A, 2hc7_A, 1k9g_A, 308d_A, 1d79_A, 1d36_A, 2nsk_A, 2des_A, 1d78_A, 6n4g_A, 5dnb_A, 461d_A, 2dpb_A, 4f3u_A, 6jv5_A, 5mvl_A, 5wv7_A, 4h5a_A, 6gim_A, 1ih6_A, 5wsp_A, 5wsr_A, 5wss_A, 4xsn_A, 2hto_B, 1d33_A, 6s7d_A, 417d_A, 6a85_A, 1m6r_A, 2hto_A, 5xk0_A, 344d_A, 6jjf_A, 388d_A, 1s2r_A, 2dpc_A, 389d_A, 2dp7_A, 1g8n_A, 4r4a_A, 5dsb_A, 1d10_A, 1d23_A, 2avh_A, 482d_A, 385d_A, 295d_A, 1d15_A, 1z3f_A, 1l6b_A, 2b2b_A, 1zf0_A, 441d_A, 2gw0_A, 1puy_A, 1imr_A, 1ikk_A, 1dcr_A, 1d9r_A, 1d49_A, 2o4f_A, 1zf6_A, 5xk1_A, 1cw9_A, 473d_A, 5mvq_A, 5mvk_A, 4p1d_A, 1jpq_A, 2hbn_A, 6ip7_A, 6m4t_A, 5w1z_A, 1zfa_A, 5zas_A, 4f2x_B, 6mc3_A, 2qef_A, 1d67_A, 6dwt_A, 1sgs_A, 1ub8_A, 1v3o_A, 1uhy_A, 1r3z_A, 160d_A, 181d_B, 137d_A, 1s23_A, 3bse_A, 3bse_B, 1j8l_A, 5dsa_A, 1zf4_A, 4ez2_A, 213d_A, 370d_A, 1xjx_A, 423d_A, 2dz7_A, 1ue4_A, 2plb_A, 2pl8_A, 368d_A, 1dnz_A, 3pa0_A, 340d_A, 115d_A, 313d_A, 6n65_A, 3gsk_A, 306d_A, 1p4y_A, 5mvp_A, 351d_B, 4dwy_B, 3qxr_A, 1i1p_B, 1i1p_A, 118d_A, 1ljx_A, 400d_A, 6dxj_A, 5iyg_A, 5ezf_A, 5ezf_B, 383d_A, 6m5j_B, 6m5j_A, 4ys5_A, 1zfb_A, 4fxm_A, 6g8s_B, 2a7e_S, 1nr8_A, 4e7y_A, 5ip8_B, 5dev_A, 5fhj_A, 5ewb_B, 5iyj_A, 5ewb_A, 5iye_A, 5cdb_A, 2d94_A, 6s15_A, 396d_A, 278d_A, 348d_A, 1xjx_B, 1d12_A, 1d90_A, 1d56_A, 196d_A, 1nvn_A, 4gre_A, 3tok_B, 4gre_B, 1zey_A, 1zfg_A, 1l1h_A, 3hr3_A, 3iff_A, 1jtl_A, 1v3n_A, 9dna_A, 341d_A, 3lpv_A, 3lpv_B, 3c2j_A, 6rnl_A, 5ls8_A, 4r47_A, 3eum_A, 4zkk_A, 6jr4_A, 1d3r_B, 6qt2_A, 312d_B, 334d_A, 6qt1_A, 1d3r_A, 159d_A, 138d_A, 6qjo_A, 184d_A, 6wck_A, 4wo2_A, 1zf3_A, 200d_A, 307d_A, 307d_B, 241d_A, 1vt8_A, 3pbx_A, 4e95_A, 158d_A, 4ymc_A, 5ua3_A, 3uyh_A, 5ccw_A, 1f6e_A, 317d_A, 4r45_A, 243d_A, 1ih4_A, 2b1b_A, 2b1b_B, 3igt_C, 110d_A, 414d_A, 327d_A, 3igt_A, 3igt_B, 399d_A, 260d_A, 254d_A, 1p54_A, 395d_A, 304d_A, 369d_A, 192d_A, 3n4n_E, 3n4n_D, 4f8i_A, 5dam_A, 4z4b_A, 4xno_A, 4h29_A, 1sm5_B, 1xjy_A, 275d_A, 1zfc_A, 1jrn_A, 1da3_A, 189d_A, 3eru_A, 238d_A, 2gwq_A, 380d_A, 5ju4_A, 5jvw_A, 2d95_A, 1dcw_A, 1sm5_A, 1uhx_A, 1d57_A, 2d47_A, 1p1y_X, 4izq_A, 3e9w_A, 3e9w_B, 239d_A, 208d_A, 2hri_A, 1d91_A, 1hq7_A, 4ocd_A, 4rip_A, 343d_A, 1d16_A, 1zje_A, 1zje_B, 1d26_A, 1d93_A, 384d_A, 1rsb_A, 5ana_A, 287d_A, 253d_A, 119d_A, 3cco_A, 2b1c_A, 458d_B, 458d_A, 188d_A, 1ilc_A, 3eqw_A, 1zjf_A, 3eui_A, 1zew_A, 257d_A, 1d92_A, 111d_A, 6mc4_A, 249d_A, 252d_A, 1lp7_B, 1lp7_A, 195d_A, 1d89_B, 1d89_A, 3t8p_A, 281d_A, 2avj_A, 3qsf_X, 1gqu_A, 282d_A, 1k8p_A, 378d_A, 3ce5_A, 3qsc_X, 358d_A, 237d_A, 1dvl_A, 1lu5_A, 6wk7_B, 206d_A, 294d_A, 1qph_A, 3l1q_A, 1n4e_B, 1pnn_B, 1n4e_A, 116d_A, 1vt7_A, 1d98_B, 1vt9_A, 2b1d_A, 1zfh_A, 3ey0_A, 117d_A, 1qp5_A, 1qp5_B, 1d31_A, 309d_A

Protein-DNA complexes, listed as pdbID_chain:

4m9v_A, 4m9v_B, 4gzn_B, 6ukf_B, 6ukf_A, 1pfe_A, 3go3_A, 6ukg_A, 6ukg_B, 4lup_B, 6kbx_A, 6dp8_C, 6dpi_C, 6dph_C, 1k3x_B, 1k3x_C, 6dog_C, 6dob_C, 6dpb_C, 6dpc_C, 6dop_C, 1xvk_A, 6do9_C, 6doh_C, 6dos_C, 2hax_C, 2vla_L, 2vla_M, 3ndh_C, 3ndh_D, 5exh_A, 3sjm_C, 3sjm_D, 4uqm_B, 4uqm_C, 4nih_C, 4nih_B, 5cl8_C, 5cl8_B, 4xzf_B, 1xvr_A, 1mnn_B, 1mnn_C, 2ea0_B, 2ea0_C, 5dlo_B, 3fde_E, 3fde_C, 4x9j_B, 4x9j_C, 1k3w_B, 4ecv_T, 5kfc_P, 5kfz_T, 5kft_P, 5kfz_P, 3jxy_B, 3jxy_C, 5cl7_C, 5cl7_B, 5mct_C, 5kl3_B, 5kl3_C, 3eyi_C, 5kfb_P, 4b21_Y, 5kfs_T, 4ecq_T, 5kfq_T, 5kg6_T, 5kfe_P, 5mg7_C, 5hf7_C, 5hf7_D, 4ed8_T, 5kg6_P, 1c8c_B, 1xyi_B, 5kfg_P, 5kfu_P, 2nq9_B, 5kfx_P, 5kfc_T, 4ecv_P, 5kfo_P, 5kfq_P, 6u17_D, 6u17_C, 4z47_D, 6bd0_B, 6bd0_C, 5kfa_T, 5kfn_P, 5kfx_T, 4b21_X, 5kfb_T, 5kfe_T, 2odi_D, 2odi_C, 2nq9_D, 4ed8_P, 5kfa_P, 5kfs_P, 3zdb_X, 4nig_B, 3o1t_C, 4nid_B, 4hly_C, 3o1t_B, 6ml6_E, 6ml4_F, 6ml4_E, 4htu_C, 6od3_W, 3gox_C, 3gox_D, 2oaa_D, 2oaa_C, 5swm_D, 5vzc_T, 1sx5_C, 6lqf_B, 1azp_B, 5dwa_C, 1bf4_C, 5inl_C, 4gz1_C, 5kg2_P, 6dsw_P, 6p1n_T, 5kg4_P, 4wcg_C, 6q6r_A, 1egw_E, 1egw_F, 1llm_A, 1wto_B, 1dfm_C, 1qum_B, 2adw_A, 1dp7_D, 4lzg_T, 6bdb_B, 6bdb_C, 5vza_T, 1wtv_B, 1xvn_A, 1bf4_B, 4s0n_E, 3o1p_C, 3o1p_B, 3pv8_C, 3pv8_B, 6j0h_A, 5tkz_C, 2r1j_B, 2r1j_A, 3jx7_C, 3jx7_B, 5cl6_B, 5cld_C, 5uuf_B, 5uuf_C, 3i8d_B, 3ey1_B, 4opk_B, 4bwj_C, 1a1i_B, 4r2a_B, 4r2a_C, 4r2q_B, 1aay_C, 6fbc_C, 6fbc_B, 6ml6_F, 4r2q_C, 4hug_C, 4opj_B, 5hnf_L, 5hnf_M, 4q8e_T, 4q8e_P, 6p0c_B, 2xhi_B, 3gpu_C, 4ofa_C, 4ofa_D, 4bwj_B, 2xhi_C, 6p0c_D, 6p0c_C, 6gce_C, 6gce_D, 5yty_A, 1qum_C, 1qum_D, 5ytz_A, 6gcf_D, 6gcf_C, 2evf_B, 2evf_C, 2evg_B, 2evg_C, 3ti0_B, 5z7i_D, 5yiw_E, 3thv_B, 6b0o_B, 6b0o_C, 6jip_B, 5hk3_C, 5odl_D, 2euz_B, 2euz_C, 4hue_C, 4dfk_B, 4c8m_B, 4c8m_C, 5uuh_C, 5uuh_B, 5dff_P, 4z3c_A, 4ez9_B, 3thv_C, 3ti0_C, 4f4k_B, 4dse_C, 4ez6_B, 4ez6_C, 4f4k_C, 4ez9_C, 4f3o_B, 3px4_C, 5dff_V, 5dff_D, 4f3o_C, 2etw_B, 2etw_C, 2eux_C, 2eux_B, 6ryd_C, 6ryd_D, 5kl7_B, 4tqr_T, 4tqr_P, 4dsf_C, 2g8i_C, 2g8f_C, 6kij_A, 4ix7_C, 6dsw_T, 6fbe_B, 6fbe_C, 5mf7_C, 5dfi_P, 6bow_P, 3pvi_C, 5dfi_V, 6bow_V, 6we9_B, 5ciy_C, 5ciy_D, 6e94_C, 6e94_D, 6nua_C, 6nuh_C, 6o19_C, 6o19_B, 2fmp_T, 4kli_T, 2fmp_P, 4kli_P, 5wo0_T, 3gq4_B, 3u6e_C, 2axy_E, 5o63_C, 3bs1_B, 3bs1_C, 4pcz_B, 4pcz_C, 5wo0_P, 2py5_D, 3s57_B, 3s57_C, 1t9i_C, 1t9i_D, 5o63_D, 6fl1_B, 1fiu_I, 4hc9_Y, 4hc9_Z, 5mcv_C, 1eon_C, 4xrm_M, 4xrm_L, 4pd2_C, 2zkd_C, 2zkd_D, 6mih_B, 6mih_G, 2p2r_B, 1eon_D, 1a1i_C, 1a1h_C, 1jk2_B, 6fl1_C, 6a8r_E, 6a8r_F, 6on0_C, 6fbq_C, 6fbq_D, 3u6p_B, 3u6p_C, 2g8k_C, 3uld_C, 1a1h_B, 5z7i_E, 6kbs_D, 3kxt_B, 3g9m_D, 3g9m_C, 2ih2_B, 2ih2_C, 6el8_B, 6el8_C, 6ipg_T, 3jr5_B, 1l1z_B, 3gpu_B, 4o3r_P, 6mu4_P, 3g9o_C, 3g9o_D, 1r2z_B, 1r2z_C, 4hik_B, 4f8r_C, 4f8r_B, 4n5s_C, 3py8_C, 5nkl_C, 4dfk_C, 5iij_P, 3d2w_B, 6cvq_E, 6eo6_D, 4lz1_D, 3sau_C, 3sau_B, 2bcq_P, 2bcr_T, 5zkm_B, 6h0s_B, 2bcq_T, 1jk2_C, 4pd2_B, 6ogk_C, 6ogk_D, 3m4a_D, 3m4a_E, 2itl_W, 2itl_C, 6kbz_A, 6dfb_D, 6dfb_E, 1l3l_E, 4dqs_C, 6iod_C, 4n5s_B, 4a75_B, 5td5_C, 6jiq_B, 3jxb_B, 3jxb_A, 5hdn_E, 4xpc_B, 6is8_C, 6is8_D, 2euw_B, 2euw_C, 6ml3_E, 6ml3_F, 6p0d_C, 6b1r_B, 6b1r_G, 4huf_C, 5zmo_B, 5kl2_B, 6od4_W, 3gq4_C, 3gpx_C, 4kb1_C, 3igl_B, 2ac0_E, 4oln_E, 4oln_F, 5ywt_C, 5v1g_P, 6bek_B, 3po4_C, 3s5a_C, 3s5a_B, 3rkq_C, 3rkq_D, 3po4_B, 5vhv_C, 5vhv_D,

1jx4_P, 5hrb_D, 1orn_C, 4c8l_C, 2gb7_F, 2gb7_E, 2heo_B, 2c7p_C, 1l1t_C, 1dsz_C, 1dsz_D, 3jr5_C,
1l1z_C, 5mcu_C, 5b2o_C, 5b2o_D, 4hio_B, 5b2p_C, 5vbs_B, 5vbs_G, 3igk_B, 4ibw_B, 4hj7_B, 4kmf_B,
5d9i_X, 2wbs_F, 2wbs_G, 2voa_C, 3py8_B, 6p0d_B, 1wtq_B, 5e41_B, 5d9i_Y, 2z70_B, 1h6f_C, 2c7p_D,
1jx4_T, 4him_B, 2bop_B, 1rh6_C, 1rh6_D, 1dc1_C, 1d2i_C, 1i3w_A, 1d02_C, 4bwm_B, 3rrh_B, 5b2q_D,
6mik_G, 3fsi_B, 5u2t_P, 5vs3_T, 6cr4_P, 6btf_P, 5vs3_P, 5ff8_D, 5o7t_B, 3u6s_B, 2ih5_B, 2ibt_C,
2ibt_B, 1zs4_U, 1zs4_T, 4ibu_E, 5b2p_D, 5v1g_T, 6kcq_A, 5v1o_P, 4klg_P, 6cr4_T, 4klg_T, 6fbg_B,
6fbg_C, 6e33_C, 6e33_B, 5hru_C, 5uug_C, 3mr5_P, 4ed7_P, 6m7p_P, 4o3s_P, 5iij_T, 5d8k_A, 5clc_C,
5clc_B, 3nci_T, 3nci_P, 3qep_T, 3qep_P, 3qex_T, 4j2d_P, 3sq1_P, 4j2a_P, 3qev_P, 3qex_P, 3i0w_C,
1xjv_B, 3i0w_B, 3g00_I, 2c62_C, 5yuy_C, 5yuy_B, 3kde_A, 3kde_B, 3g00_H, 5kkq_B, 5kkq_C, 4klo_P,
4klm_T, 4klj_P, 5u2r_P, 4klm_P, 4yd1_T, 5iii_P, 3fdq_C, 3fdq_D, 1ztt_B, 4c8o_C, 3jxz_B, 3jxz_C,
2vbl_T, 5hre_B, 6d0z_P, 2bcr_P, 3mr3_T, 4y60_A, 4y60_B, 3oqg_C, 3oqg_D, 6d0m_T, 3mr3_P, 2o4a_B,
2o4a_C, 6m7p_T, 6d0z_T, 6bce_B, 6bce_C, 6p0d_D, 4r63_T, 5zyu_C, 3jy1_C, 5clb_C, 3qev_T, 3si6_T,
4j2d_T, 3mfi_P, 3mfi_T, 1suz_C, 5f8a_C, 5wx9_B, 5wx9_C, 5clb_B, 3h8r_B, 3h8r_C, 4i2o_W, 4i2o_X,
1zzi_C, 3vke_R, 3u6s_C, 5yuz_B, 6jum_B, 2es2_B, 1l1t_B, 3gq3_B, 4g4n_B, 3gpy_B, 6rar_D, 6rar_B,
6rar_C, 6ctq_T, 4klf_T, 4klj_T, 6p0e_D, 4e9f_C, 6ctr_T, 5u9h_T, 4kll_T, 5dgb_P, 6nkv_T, 4klo_T,
6lff_C, 4ljr_C, 3q23_C, 2fjx_B, 4lb6_C, 6asd_A, 4elv_C, 3mr5_T, 6ccg_C, 3qno_T, 4dtj_T, 4j2a_T,
5iii_T, 4qwb_B, 4qwb_C, 3bam_C, 3bam_E, 1lmb_1, 1lmb_2, 2bdp_T, 4bdp_P, 6b1q_G, 2wiw_C, 3rrh_C,
4dlg_C, 3t3f_C, 5o7t_C, 4dih_D, 1a73_C, 1a73_D, 1g9z_F, 4zsf_B, 1u1r_B, 1ssp_B, 1j75_B, 4jcy_C,
4jcy_D, 4g92_D, 4g92_E, 5w9q_C, 3v9z_C, 4tup_T, 4r63_P, 4elu_C, 1g9z_C, 1g9z_D, 1g9z_E, 4bdp_T,
1u1q_B, 2vbn_C, 2vbl_C, 2vbl_S, 2vbo_C, 2vbo_E, 1q3f_C, 1ssp_A, 2bdp_P, 2fjx_G, 1vto_C, 1vto_D,
1qna_C, 1qna_D, 3i0x_C, 1m5r_C, 1m5r_E, 3d0p_B, 1w0u_C, 1w0u_D, 1u1p_B, 1q3f_B, 3kjp_B, 1sxq_E,
1sxq_C, 1wtr_B, 1tez_I, 1tez_J, 1nkp_F, 1emh_B, 6s48_C, 1gtw_D, 1gu4_D, 2e42_D, 2e42_C, 1wtp_C,
5wvw_C, 5e41_C, 3ojs_C, 4r55_B, 5dfj_V, 6gcd_C, 6gcd_D, 6w0q_V, 5dfj_P, 6bor_P, 5dg0_P, 6ogj_C,
5szt_C, 6fbh_B, 6fbh_C, 5hrd_E, 3u6c_C, 1ytb_C, 2evi_B, 2evi_C, 6bor_V, 6wea_E, 2ih5_C, 2dp6_C,
2dp6_D, 1cl8_B, 3d0a_E, 1ztt_G, 5deu_B, 5deu_C, 5kn8_C, 5kn8_D, 3q24_C, 6d92_G, 6d92_J, 6lrd_C,
5bua_B, 6j80_C, 6b0r_B, 6b0r_C, 6df5_D, 6dfa_E, 6df5_E, 3sq1_T, 5l1k_P, 4hid_B, 4o6a_C, 5xzg_F,
5xzg_E, 3gq3_C, 5yv2_C, 5yv2_B, 5yuu_H, 2gig_E, 5chz_C, 3mr2_P, 5yuz_C, 6d0m_P, 6blw_B, 6blw_C,
3qew_T, 3si6_P, 3qno_P, 3ngi_P, 4dtj_P, 3qew_P, 4m3z_P, 3ngi_T, 4dtu_T, 4m3z_T, 4dtx_T, 4m3y_P,
4fjl_P, 4dtu_P, 4dtx_P, 6c1t_B, 6p0e_B, 5tbc_P, 4f5p_P, 3ogu_P, 3ogu_T, 5inp_C, 5inq_C, 4dwi_B,
2ofi_B, 2ofi_C, 3v6t_G, 3v6t_H, 4rdu_B, 2ahi_E, 5wm1_T, 5wm1_P, 4gjr_G, 4gjr_H, 1zbi_D, 4gz2_C,
4hj9_B, 4j9l_T, 4aik_D, 2w7n_E, 2w7n_F, 2c7q_C, 2c7q_D, 6p0e_C, 6q1v_C, 6dfa_D, 6iiq_D, 6d95_C,
5trd_G, 6q1v_D, 5v1h_P, 5e69_C, 5e69_D, 3u6o_C, 6q1v_B, 3go8_C, 4g4n_C, 2opf_B, 6dfc_D, 6dfc_E,
2oxv_C, 1ckq_B, 4m9e_B, 4m9e_C, 6uwj_B, 6uwj_C, 6bux_B, 4o6a_E, 4m3y_T, 2jej_P, 2jej_T, 5hp4_X,
1qne_C, 1qn3_D, 1qn5_C, 1qn5_D, 1qn3_C, 1qne_D, 1qn4_C, 1qn4_D, 2i0q_D, 1jb7_D, 3l2c_B, 3l2c_C,
4m45_T, 3s9h_T, 3qnn_T, 4fjl_T, 4l5r_A, 5hnh_M, 5hnh_L, 4dts_T, 4fj9_T, 3qer_T, 4fj7_T, 4dtm_T,
3uiq_T, 3s9h_P, 4dts_P, 3sm4_D, 3sm4_E, 4c8n_C, 6bda_B, 6bda_C, 6bda_D, 6bda_E, 3imb_E, 5haw_Z,
5t01_C, 5t01_D, 2g1p_F, 6w0q_D, 2euv_B, 2evj_C, 2evh_B, 2evj_B, 3imb_F, 2asd_D, 2asd_E, 3rax_E,
3rax_D, 5yus_B, 3dvo_E, 5yuu_B, 3ga6_F, 4f4w_E, 4f4w_F, 3g6t_D, 3g6t_C, 3g6u_D, 3g8u_D, 3vaj_E,
3vk8_D, 4kaz_B, 4fk2_T, 4fk4_T, 4lvi_C, 4roc_N, 4roc_T, 3vk8_C, 5iil_T, 1esg_D, 2h7g_Y, 4ejy_D,
2f5p_C, 2xo6_B, 3jxc_A, 1mus_B, 1mus_C, 6s48_E, 2c7r_C, 2c7o_D, 5hto_C, 5hto_F, 4xqj_B, 4xqj_C,
5iff_C, 5iik_T, 3hwt_P, 4glx_B, 4glx_C, 4k1g_F, 4k1g_H, 1e3o_A, 1e3o_B, 6vdd_C, 5iim_T, 2xqc_C,
3aaf_C, 2vjv_C, 4xa5_P, 4b22_Y, 2w42_P, 2w42_Q, 6cc8_C, 2a07_B, 2a07_A, 3sar_C, 3v9x_E, 3pnc_B,
3mr6_T, 3mr6_P, 4k1g_E, 6s48_G, 5iim_P, 4b23_X, 3bdp_T, 2ht0_F, 4b22_X, 2vbn_T, 2ve9_J, 2ve9_I,
1xsn_T, 5iil_P, 2bcv_P, 3mdc_P, 2c7r_D, 2c7o_C, 1wte_Y, 1wte_X, 1puf_D, 1puf_E, 1qn9_C, 6ueo_C,
1qn9_D, 2vjv_E, 1mw8_Y, 1lat_C, 1oe4_F, 1p71_C, 1wd0_B, 1y8z_C, 1tro_I, 1nh2_E, 1nh2_F, 1eo4_C,
1eo3_C, 1esg_C, 1b97_C, 1b94_C, 1cdw_B, 1cdw_C, 6qec_U, 6qec_B, 6rce_D, 5zfz_D, 5zfz_E, 6j9b_B,
6j9b_C, 5k07_B, 5d49_B, 6a47_C, 6vdd_B, 3osn_B, 6nkw_T, 6fbi_C, 6fas_C, 6fas_D, 3upq_P, 3u6l_C,
3sar_D, 2pfo_P, 1y8z_D, 2nll_C, 2nll_D, 2og0_C, 2og0_D, 1oe4_E, 6vdd_F, 2h7g_Z, 2hdd_C, 2hdd_D,
4glx_D, 2b0e_C, 2pfn_P, 2xo6_C, 1bnz_B, 2np7_C, 1a1k_B, 1a1k_C, 4hn5_C, 4hn5_D, 4hje_E, 4hje_F,
4u0y_E, 3mq6_K, 5kvy_C, 3dpg_C, 3spd_E, 3spd_F, 6mu5_P, 4tu8_E, 4p4m_E, 3m7k_B, 3ldy_C, 3bep_C,
3bep_D, 3k59_P, 3k59_T, 3m7k_C, 4a76_B, 5wm8_P, 5wm8_T, 5egb_C, 5egb_D, 5ei9_A, 5ei9_B, 6mg2_C,
1bc8_A, 1bc8_B, 2gij_E, 5kn9_C, 3m9o_T, 3m9o_P, 3m9n_P, 1cyq_C, 6g1t_E, 6g1t_D, 1kx5_J, 1kx5_I,
3f8j_G, 3eeo_D, 4gjp_G, 4gjp_H, 3f8j_F, 3eeo_C, 4osr_G, 4osr_H, 6rce_B, 3mda_P, 3q8p_T, 5zkl_B,
3h8o_C, 3btx_B, 3h8x_C, 3h8o_B, 4jrp_C, 3cmy_B, 3cmy_C, 3h8x_B, 4uno_C, 4uno_B, 3hwt_T, 5zoe_B,
4j9s_T, 5d23_B, 5vaj_D, 3hw8_B, 1omh_B, 1lv5_D, 1owf_C, 2ht0_E, 1owf_E, 5orq_B, 5zoe_C, 5d49_C,
4yoy_B, 1sa3_C, 2han_C, 2han_D, 2fjw_G, 2dem_D, 4j9n_P, 4dtn_T, 2hr1_D, 2hr1_C, 4ou6_L, 5ulx_T,
2zo1_E, 6mht_C, 2i13_C, 2i13_D, 4dl7_T, 4dl7_P, 3ldy_B, 1sfu_C, 5j5p_C, 5d9y_B, 5d9y_C, 5d4s_T,
5d4s_U, 5zg9_C, 4h0e_U, 4h0e_T, 4usg_C, 3vd6_A, 3vd6_B, 4lox_B, 4lox_C, 4lox_D, 4lox_E, 5npk_E,
5iwi_F, 5iwi_G, 5iwi_E, 4fjm_T, 6dsy_T, 3ne6_T, 6dsx_T, 6dsu_T, 6dsy_P, 3u6q_C, 5iik_P, 3g6p_D,
5cby_D, 3g6p_C, 1s32_I, 5y0d_I, 1kx3_I, 2evh_C, 5ke6_B, 5ke7_C, 6rau_C, 6dsv_T, 5ke6_C, 4tu9_E,
5cby_C, 4ff3_C, 4rtq_G, 4rtq_F, 3osg_B, 3osg_C, 4f4x_T, 3qz8_T, 4f4x_P, 3qz7_T, 3qz8_P, 5xh6_D,
3iay_P, 3iay_T, 4ebe_T, 3gv5_E, 3gv5_F, 3gv8_T, 1du0_C, 1du0_D, 3vxv_C, 3vxv_B, 1xo0_C, 1xo0_D,
3c2p_C, 3btx_C, 3cbb_C, 3cbb_D, 3cvv_C, 3cvv_D, 4lnq_C, 2dnj_C, 1w0t_C, 1w0t_D, 4qz9_D, 4lb5_C,
4rkh_B, 4rkh_A, 4tqs_D, 4fbt_T, 4fbt_P, 1stx_C, 1rys_C, 3exj_C, 3exj_D, 1g9y_D, 1g9y_C, 2wq7_C,
2wq7_D, 3cvu_C, 2uvw_T, 2imw_S, 2bq3_T, 3ted_C, 3ted_B, 4cn5_C, 4cn5_D, 6u7t_B, 4dl3_P, 4dl4_P,
4dl4_T, 1a3q_D, 5n2q_B, 1bc7_A, 1bc7_B, 4hcb_C, 5b2r_C, 5b2r_D, 5xh6_C, 5awh_D, 2oyt_B, 3wvk_F,
5fgp_B, 1s10_B, 3nae_T, 4ea4_C, 4ejy_C, 1jxl_P, 3uby_C, 1s9f_E, 3mfh_T, 2fld_C, 2fld_D, 3mqy_D,
1t9j_C, 1t9j_D, 4b23_Y, 1nlw_F, 4qeo_C, 4qen_D, 4qeo_D, 4tqs_C, 1s10_C, 3o3g_C, 4qr9_C, 4ff4_C,
1s9f_I, 3pvv_C, 3pvv_D, 2r2r_B, 2uvw_P, 2bcv_T, 2uyc_C, 2uyc_D, 3oha_T, 3ohb_T, 5hlk_E, 1qn8_C,
1qn6_C, 6uep_C, 6ueo_B, 2f5p_D, 3vk7_D, 1n3f_D, 1n3f_C, 1s0o_C, 1ua1_B, 1ua1_C, 1unm_A, 6u7t_C,
6q0c_B, 1u1k_B, 2dnj_B, 2up1_B, 1u1m_B, 1u1n_B, 1emj_B, 3mqy_C, 1fjl_D, 1fjl_E, 1fjl_F, 1gd2_A,
1bsu_C, 1g2f_A, 1g2f_B, 3zp5_G, 5hrg_C, 1bgb_C, 1jgg_C, 1jgg_D, 5yeg_D, 5yeg_C, 5yws_C, 5zvb_C,
5xh7_C, 5xh7_D, 3ndk_T, 6cst_C, 6cst_D, 6d9k_G, 5gnq_S, 5fhd_C, 2oyt_C, 6q0c_C, 1yo5_A, 1yo5_B,
2noh_C, 2bam_C, 2bq3_P, 1rxw_C, 1rxw_B, 1uut_C, 2ht0_C, 2ht0_D, 2aor_C, 1b95_C, 2d5v_C, 2d5v_D,
5wvy_C, 1po6_B, 1u1l_B, 1rvc_C, 2e52_E, 5hlk_C, 4pw7_C, 4pw7_D, 6fbf_B, 6fbf_C, 4qen_C, 5vmx_D,
5vmy_E, 6jvy_B, 4kb0_C, 4b5f_V, 4b5f_U, 3khl_D, 3khr_D, 1lwy_E, 3khr_E, 3oym_C, 3oym_D, 4e3s_T,
4fj5_T, 4fjg_T, 6fk5_B, 5o6b_C, 1rys_D, 2kfn_B, 2kfz_B, 5yuv_B, 2pyj_J, 2pyj_K, 4j9k_P, 3q8q_T,
3osf_B, 3osf_C, 4m3r_T, 3k5m_T, 3k57_P, 1kfs_B, 4hj8_B, 4hj5_B, 5yux_H, 2xsd_B, 5vu9_T, 5vu9_P,
4qwd_D, 5jre_I, 3k58_T, 2vs7_B, 2vs7_C, 4qwd_C, 5mey_D, 5eh2_A, 4gck_W, 2imw_T, 4dto_T, 4esj_C,
4qza_D, 1f44_M, 1f44_N, 6go6_F, 1qn6_D, 1qn8_D, 6uep_D, 6mht_D, 1i7d_B, 3qlp_D, 6jnm_D, 6jnm_C,
3kqn_E, 6p09_C, 6u6z_E, 6od5_E, 6fj5_F, 5vpe_E, 5vpe_F, 7cc9_E, 7cc9_D, 5v3j_A, 5v3j_B, 4cn2_A,
4cn2_B, 5d8l_A, 5d8l_C, 5d4r_T, 5d4r_U, 6t21_C, 6t21_D, 6b0p_B, 6b0p_C, 5t2n_C, 3k57_T, 1d8y_B,

3g6x_T, 2pfp_P, 5jlw_B, 5jlw_C, 3v9s_C, 4qzb_D, 6q02_P, 6q02_T, 3q8s_T, 5v3m_A, 5v3m_B, 5omf_P, 3ugo_B, 3o9x_F, 3o9x_E, 3vk7_C, 3co6_A, 3co6_B, 1ewn_D, 1ewn_E, 3ulp_Q, 3khl_E, 3s8q_C, 3s8q_D, 3sbj_C, 4is1_B, 3uk3_B, 4q0w_C, 4q0w_D, 3a46_C, 3igc_D, 1wb9_F, 2xcs_E, 3sq2_T, 3sq2_P, 2vs8_B, 2vs8_C, 2vs8_D, 2vs8_E, 4dl3_T, 4qwa_D, 3e40_E, 5jk0_E, 5jk0_F, 3c5g_T, 2w36_C, 4z5h_B, 4py5_C, 4hja_B, 2r2r_G, 1rzt_B, 1k4t_B, 1k4t_D, 1by4_E, 1pue_A, 1pue_B, 1mjo_F, 1s0o_D, 1k61_E, 1k61_F, 1jxl_T, 1u8b_B, 1u8b_D, 1u8b_E, 4qju_C, 1ph6_D, 1wb9_E, 1a3q_C, 1nvp_F, 1nvp_E, 1dux_A, 1dux_B, 1by4_F, 1j1v_B, 1j1v_C, 1fyl_C, 1ais_C, 1ais_E, 1gu5_C, 1gu5_D, 1a31_D, 1a31_C, 6kcs_B, 5uc6_B, 6d8p_G, 5k17_C, 4qtj_B, 4qtj_C, 6ur9_B, 6ur9_C, 1x9m_C, 1x9m_D, 2p0j_D, 6wc2_E, 6wc2_F, 3igc_B, 1zrf_W, 1zrf_X, 4qwe_D, 2ddg_D, 2e1c_B, 2e1c_D, 1qbj_D, 1wd1_B, 5krb_A, 5k97_E, 5k97_F, 5k97_D, 5zfw_D, 5zfw_E, 5kse_E, 3q8k_F, 2w35_D, 3nh1_E, 2bcu_P, 4gz0_C, 5mhj_E, 1je8_C, 6e8c_C, 2pqu_E, 3e3y_E, 5fq5_C, 5fq5_D, 5fq5_E, 5lxu_U, 5lxu_B, 3kmd_E, 3l8b_C, 3lap_G, 3lap_H, 4dkj_B, 4dkj_C, 4yhx_B, 4yhx_C, 4flw_P, 4flw_T, 2w35_C, 5v07_A, 5v07_B, 3si8_T, 4z5d_D, 4z5d_C, 5t8d_B, 5t8d_C, 3si8_P, 6kks_B, 6kks_C, 4dl2_P, 6d8f_G, 6fb0_D, 6fb0_F, 5ink_C, 6fke_B, 3qx3_C, 3qx3_D, 3vag_E, 3vai_E, 4lvl_C, 4lvj_C, 2xc9_T, 2jef_T, 3c1b_I, 1n48_B, 2ibk_D, 2uvv_T, 2br0_T, 3a4k_F, 2xc9_P, 2uvv_P, 2jef_P, 2br0_P, 4khq_T, 3lwm_C, 4ncb_C, 4ncb_F, 4ncb_H, 4ncb_D, 4lg0_C, 4lg0_D, 4fj8_T, 1n6j_C, 3p57_F, 3p57_E, 5eea_C, 5eea_E, 2a66_C, 2a66_B, 5l0m_C, 5l0m_B, 4mhg_C, 4mhg_B, 3g6v_T, 3c0w_C, 1r7m_C, 4crx_C, 5bt2_B, 5bt2_C, 3clz_E, 3clz_F, 3coa_B, 3coa_A, 3c29_D, 3qi5_D, 4noe_F, 4lmg_E, 4lmg_F, 1qrv_C, 4m8o_P, 4m8o_T, 4hqu_C, 3g6r_C, 4roe_T, 4roe_N, 3khc_C, 3a46_D, 1rrq_C, 2x6v_C, 2x6v_D, 3k0s_E, 3k0s_F, 3jtg_C, 3c29_C, 5a0w_B, 5a0w_C, 4dav_D, 4e0j_C, 4e0g_D, 4e0g_C, 4e0p_D, 3dsd_C, 4aqx_C, 4aqx_F, 5keg_B, 5b2t_C, 3mva_D, 3mva_E, 3h4d_T, 2i06_B, 2i06_C, 3q0b_A, 4jbm_R, 2xrz_C, 2xrz_D, 3gv7_T, 4rzr_C, 1qsl_B, 3v1z_C, 5d2s_B, 4qcl_C, 3c0w_B, 5n8r_C, 3pmn_C, 2rdj_C, 2rdj_F, 2rdj_D, 2bcs_T, 2cgp_C, 3c0w_D, 1kbu_D, 3c29_E, 1r71_E, 1r71_F, 1ma7_C, 1mey_A, 1mey_B, 1mjm_C, 1n48_C, 1n6j_D, 1p47_C, 1p47_D, 4yoq_B, 1e3m_F, 1e3m_E, 1tk5_P, 1sl2_T, 1skw_P, 1skm_D, 1tk0_P, 1tk5_T, 1tsr_E, 1ewq_C, 1ewq_D, 2dgc_B, 3qi5_C, 1dmu_F, 1i3j_B, 1i3j_C, 1ig7_B, 1ig7_C, 1b3t_C, 4aqx_E, 3f21_D, 3brd_B, 1jnm_C, 1kbu_C, 5b2s_C, 6jbx_C, 6jbx_D, 6jw0_C, 6oov_C, 6fb5_D, 6fb5_F, 5e67_B, 5e67_C, 2ex5_X, 2ex5_Y, 2pyl_X, 2pyl_Y, 2is6_C, 2irf_A, 2irf_D, 4yoq_C, 1t7p_P, 1t7p_T, 1xsl_C, 2aoq_B, 2ata_E, 4r56_C, 3lwi_C, 3igm_C, 5wvz_C, 1oup_C, 1ca6_B, 1wtw_B, 1ca5_B, 5hr9_D, 3q8k_E, 4o0i_B, 3vxx_C, 4pw5_C, 3g73_C, 3g73_D, 6t22_C, 6t22_D, 4f50_P, 4f50_T, 5npp_E, 5npp_A, 4abt_E, 6uwg_B, 6uwg_C, 3sq4_T, 3sup_P, 1h8a_D, 1h8a_E, 1qnb_D, 1qnc_C, 1r0o_C, 1r0o_D, 3wph_B, 3wpi_B, 4n41_D, 4n41_C, 1r7m_D, 3bty_B, 3bty_C, 3raq_D, 4j9m_T, 1bg1_B, 3h40_T, 3v7j_P, 3v7j_T, 1m5x_C, 1m5x_D, 5d8c_C, 5d8c_D, 2c9l_A, 2c9l_B, 1k78_C, 1k78_D, 4qze_T, 1ign_D, 1ign_C, 5xfp_C, 6us5_C, 6ur4_C, 2ssp_A, 2kzz_B, 2ibk_E, 6d8a_G, 6f58_C, 3ikt_C, 1s6m_B, 5fmp_C, 5fmp_D, 2bqr_T, 1rrj_C, 2b9s_C, 2b9s_E, 5cp2_T, 3v7k_T, 3v7k_P, 4k8z_T, 4k8z_P, 5mhk_F, 1ryr_C, 3t5h_B, 3s6i_B, 4k8x_P, 2r8i_T, 6eko_E, 3mgv_E, 3mgv_F, 1yf3_C, 1yf3_D, 4wu4_G, 4wuh_H, 6go5_G, 1nop_D, 3nh2_C, 4hqe_C, 5zog_B, 5emc_D, 3g2d_I, 3g2d_G, 3c0x_B, 3c0x_C, 3wu1_C, 3wu1_D, 3slp_D, 4ir1_H, 2wt7_C, 2wt7_D, 3mxb_C, 3mxb_E, 2xma_C, 4e0j_D, 1muh_B, 5emc_C, 3g6r_D, 5w6k_C, 4owx_L, 1r2y_C, 4jv1_B, 4fly_T, 1oup_E, 4l18_C, 3wtt_E, 2wq6_C, 4egz_T, 4egz_U, 4z59_B, 4wu4_H, 4b24_Y, 1f6o_E, 3c0x_D, 1cw0_O, 3po5_C, 4fly_P, 3mxa_C, 3mxa_D, 3mxa_E, 3mxa_F, 5ywv_C, 3px7_C, 1rrj_B, 1qtm_C, 1qsy_C, 1qss_C, 1rio_U, 1rio_T, 1lq1_E, 1lq1_F, 2hof_C, 1le8_C, 1le8_D, 1ph8_D, 1ph5_D, 1ph4_D, 1ph3_D, 1ph4_G, 1t3n_T, 1s97_E, 1skr_T, 1tk0_T, 1cw0_M, 1a6y_C, 1a6y_D, 1f6o_D, 1am9_E, 1am9_F, 1au7_D, 1au7_C, 2i3p_D, 2i3p_C, 1bl0_C, 1bl0_B, 1nfk_C, 1ma7_D, 3wvp_M, 5zfy_E, 5zfy_D, 5zva_C, 1d1u_B, 4ir9_H, 6ir8_B, 6ir8_C, 3ktq_C, 2ktq_D, 5tgx_I, 5e01_C, 5x2g_C, 5x2g_D, 5x2h_C, 5x2h_D, 1xsl_B, 2nl8_W, 2h7h_X, 2h7h_Y, 1ph3_G, 2i3q_C, 2i3q_D, 2owo_B, 1zet_T, 1zg5_C, 1zg1_C, 1zg1_D, 1zzj_D, 1qaj_C, 2geq_C, 2h27_B, 2h27_C, 1qai_C, 1ph8_G, 4j3n_C, 1a1l_C, 4egy_T, 4egy_U, 5z6z_D, 5z6z_E, 5cm3_C, 3g9j_C, 6kdm_E, 6kdn_E, 4f4z_C, 6qtk_E, 2pi0_E, 2pi0_F, 2aq4_T, 4eey_T, 5th3_I, 5th3_H, 5th3_h, 5th3_i, 4f4y_C, 4cn7_C, 4cn7_D, 6u81_C, 6pbd_X, 6pbd_Y, 4iem_F, 3gij_D, 3b39_C, 4cn3_E, 4cn3_F, 4xr1_B, 4xr1_C, 4dwp_C, 4dwp_D, 5t7x_D, 5t7x_C, 1b72_D, 1b72_E, 1pvp_C, 1pvp_D, 1mwi_D, 1f2i_A, 6go5_H, 6bse_C, 4kyw_C, 3szq_C, 4f43_C, 4lez_E, 1b8i_C, 1b8i_D, 5hhh_P, 6qtp_E, 4un5_C, 4un5_D, 4be2_C, 4be2_D, 5v0a_B, 5uzv_A, 5v0a_A, 4iem_G, 6pz3_P, 6pz3_T, 4osk_G, 4osk_H, 4q0z_C, 4q0z_D, 5bs8_E, 5bs8_F, 3h0d_C, 3h0d_D, 3vyb_C, 2gws_G, 4tmu_B, 1ozj_D, 3hxo_B, 3g0r_G, 3g0r_K, 5jjv_C, 5dwb_C, 3t5j_B, 2vju_C, 3maq_T, 3mln_C, 4wul_H, 4wul_C, 1hcq_C, 1jj4_C, 2c6y_C, 2c6y_D, 3od8_I, 3od8_J, 1qln_N, 1qln_T, 1mjq_E, 1mj2_F, 1mow_B, 1mow_C, 1n56_D, 1ku7_B, 1ku7_C, 1trr_C, 1hcq_D, 6s6h_C, 1ga5_D, 1cez_T, 1cez_N, 1d3u_C, 1d3u_D, 1crx_E, 3vah_E, 1ga5_C, 1jmc_B, 5zjt_D, 5zjq_C, 5zjt_C, 5xuz_C, 6k2j_E, 6k2j_F, 4nca_G, 4ux5_C, 1sxp_C, 1sxp_D, 2ezv_F, 2qhb_D, 2ntc_W, 2ntc_C, 1s97_I, 2gws_F, 2hof_D, 3mip_D, 3qqy_B, 4h10_C, 4h10_D, 2isz_E, 3m9e_C, 3bjy_T, 1ihf_E, 6wya_C, 6wya_B, 3vwb_B, 5v3g_B, 6fqp_L, 5fd3_C, 5fd3_D, 5j2m_P, 5j2p_T, 5j2m_T, 5zjq_D, 6kqf_G, 3tq6_C, 3tq6_D, 6fb8_D, 5cdp_G, 5h9f_N, 4gct_W, 2nqj_C, 2nqj_D, 5cdp_E, 2bnw_E, 2bnw_F, 1pa6_D, 1pp7_E, 1pp7_F, 1tc3_A, 3qe9_B, 6fb8_C, 1pa6_G, 6dg0_C, 5ity_D, 3l4j_D, 3l4j_B, 4bdy_D, 5j2p_P, 6anq_F, 6an8_F, 6an8_E, 6avm_F, 6ujy_T, 4un3_C, 5fw1_D, 3c25_C, 3c25_D, 3crx_D, 3rn5_K, 4bul_E, 3e54_E, 1tx3_E, 3ebc_E, 5a74_C, 5a72_C, 5cdm_I, 3gp8_X, 4bul_F, 5iwm_F, 6fwr_B, 3zvk_X, 3zvk_Y, 3qyn_E, 4f41_C, 3us0_E, 4z5c_C, 4z58_B, 4z5c_D, 4aae_G, 4aae_E, 4aaf_E, 5w2c_C, 5w2c_D, 1r4o_C, 1n3e_C, 4aab_E, 2gie_E, 1ubd_A, 1ubd_B, 1bdt_F, 1bdt_E, 1per_B, 1t2t_B, 1t2t_C, 1u0c_C, 1u0c_D, 1hlv_B, 1hlv_C, 1j3e_B, 1j3e_C, 1f4k_E, 1f4k_D, 1diz_C, 3e54_C, 1akh_C, 1akh_D, 5a74_E, 1ckt_B, 1ckt_C, 1a35_C, 1a35_D, 1j59_C, 1j59_D, 1kc6_E, 6ki6_C, 6ki6_D, 6lbr_C, 6emy_D, 6k8o_B, 5u01_E, 6pax_B, 6pax_C, 4aab_D, 3c28_D, 1yrn_C, 1yrn_D, 2o5i_G, 2o5i_I, 1qps_N, 1rm1_D, 1rm1_E, 2crx_C, 1zme_A, 1zme_B, 1qri_M, 1qrh_M, 1eri_B, 9icx_T, 1xhv_F, 1xhv_E, 2alz_T, 5cdm_E, 4izz_C, 3ubt_C, 4e10_C, 4e10_D, 3d1n_A, 3d1n_B, 3e44_F, 3e44_E, 3vyq_B, 3vyq_C, 1t8e_D, 3vea_M, 2ypf_B, 2ypf_C, 5d5v_A, 5d5v_C, 2qk9_C, 4u6p_C, 4un3_D, 6fi8_E, 4da4_C, 4da4_D, 5czz_D, 6fqv_E, 4fbu_C, 3zhm_B, 3zhm_C, 4aa6_D, 2r5y_C, 2r5y_D, 1huo_D, 1rr8_A, 1mnv_A, 1ej9_C, 1ej9_D, 1huz_D, 1huo_C, 2r5z_C, 2r5z_D, 1gt0_A, 1cf7_D, 1cf7_C, 9icw_T, 5yi2_C, 2gli_C, 9icw_P

Ensemble of the analyzed DNA structures containing 2,727 DNA chains, 48,401 dinucleotides, and 343,875 associated water molecules.

	A_A	A_T	A_C	A_G	T_A	T_T	T_C	T_G	C_A	C_T	C_C	C_G	G_A	G_T	G_C	G_G	sum
AA00	321	618	1,100	1,111	739	463	993	693	939	1,182	3,326	2,103	464	1,758	2,725	2,549	21,084
AA01	9	36	103	390	276	51	57	107	123	94	160	516	498	24	391	976	3,811
AA02	989	707	175	77	802	1,013	363	131	159	277	509	354	55	170	185	30	5,996
AA03	0	4	10	0	0	0	0	0	0	3	21	18	0	9	18	16	99
AA04	2	31	90	40	89	18	19	47	6	49	101	65	30	22	79	289	977
AA05	0	0	0	0	29	0	0	0	0	0	0	0	0	0	0	0	29
AA06	5	3	3	3	0	0	0	0	0	0	2	13	0	8	25	135	197
AA07	0	0	0	0	0	0	0	0	0	0	4	0	0	0	0	0	4
AA08	36	21	130	98	16	9	69	102	10	62	157	45	16	14	317	134	1,236
AA09	0	0	9	0	14	0	10	0	9	0	27	15	2	0	9	0	95
AA10	0	0	7	2	16	0	5	0	18	0	0	18	0	7	0	37	110
AA11	0	0	0	0	0	0	0	0	0	0	1	0	0	0	0	0	1
AA12	0	0	7	9	0	0	12	0	18	0	12	69	6	5	0	14	152
AA13	0	0	0	0	0	0	0	0	0	0	0	0	0	0	0	0	0
AAS1	0	0	0	0	0	0	0	0	0	11	0	0	3	0	0	0	14
AB01	177	598	357	415	1,175	842	612	994	1,486	1,391	813	3,775	315	433	890	422	14,695
AB02	13	97	15	31	24	6	12	54	5	29	30	51	14	7	18	111	517
AB03	50	135	112	98	309	238	276	381	398	303	285	796	106	45	249	120	3,901
AB04	13	104	79	385	612	38	14	207	53	224	78	80	40	334	103	363	2,727
AB05	144	406	300	346	317	283	87	301	278	556	559	691	187	1,205	262	581	6,503
AB1S	0	0	0	167	0	0	0	16	2	0	0	28	0	0	0	0	213
AB2S	0	0	0	0	0	0	9	0	0	0	0	16	0	0	0	0	25
BA01	130	403	845	131	41	233	227	62	115	286	846	268	237	488	1,409	287	6,008
BA05	1,053	3,542	2,717	1,319	562	1,025	1,933	234	774	794	7	3	726	1,564	3,456	1,243	20,952
BA08	73	84	263	656	123	37	232	150	111	28	199	562	291	126	1,057	309	4,301
BA09	25	294	335	104	22	0	15	67	12	42	0	8	86	24	103	258	1,395
BA10	18	13	201	80	245	62	88	101	70	0	49	44	27	98	185	102	1,383
BA13	147	67	100	131	23	0	109	88	78	10	31	8	245	142	141	131	1,451
BA16	90	24	11	62	17	10	35	52	21	0	0	14	311	12	126	70	855
BA17	22	33	27	33	44	104	44	18	70	0	71	80	24	34	114	5	723
BB00	8,838	6,148	4,496	7,208	7,082	7,866	6,567	8,111	5,529	5,126	5,235	8,275	8,909	7,766	7,612	10,897	115,665
BB01	3,159	4,388	1,783	1,622	1,513	5,483	3,746	1,509	835	2,381	1,936	1,041	728	2,615	1,952	1,213	35,904
BB02	540	850	485	316	214	621	261	180	151	183	250	82	221	366	379	390	5,489
BB03	21	61	308	42	38	173	73	1	12	15	46	13	11	44	79	58	995
BB04	1,365	205	254	1,372	1,097	121	348	1,615	1,089	333	464	2,469	1,700	282	936	1,936	15,586
BB05	5	3	119	83	51	4	9	0	10	8	3	0	18	16	21	12	362
BB07	954	65	107	889	1,958	52	152	2,293	2,413	253	529	2,834	2,010	164	1,884	3,474	20,031
BB08	4	22	31	7	55	2	3	44	4	15	6	65	20	269	56	47	650
BB10	55	20	7	23	28	9	19	14	49	14	78	114	39	29	34	35	567
BB11	13	15	31	12	8	65	23	30	36	14	29	89	8	4	29	26	432
BB12	282	320	757	29	5	55	238	13	1	9	57	6	271	145	304	65	2,557
BB13	23	8	19	16	7	9	17	4	4	17	0	6	7	30	44	4	215
BB14	4	5	0	1	0	2	1	4	5	0	0	1	3	0	0	4	30
BB15	16	83	186	39	65	84	160	24	25	42	165	43	50	78	155	111	1,326
BB16	183	60	44	64	358	74	64	229	304	268	457	350	87	227	136	111	3,016
BB17	0	0	0	0	0	0	0	0	2	1	0	3	13	0	2	29	50
BB1S	0	0	0	0	0	0	0	0	0	0	0	0	0	0	0	290	290
BB20	0	0	0	0	0	364	0	0	0	0	0	0	0	0	0	0	364
BB2S	0	0	0	0	3	0	0	0	0	0	0	4	0	0	0	539	546
BBS1	218	151	72	348	17	29	0	0	5	0	0	0	95	34	16	2,179	3,164
IC01	30	0	0	0	0	11	0	106	0	19	0	38	13	8	0	0	225
IC02	0	0	0	0	0	67	0	0	0	0	0	0	0	0	0	0	67
IC03	0	0	0	0	0	0	0	0	0	0	0	14	0	0	0	0	14
IC04	0	10	0	32	0	0	0	0	0	11	11	41	0	0	2	0	107
IC05	0	0	0	0	22	0	0	0	40	0	0	263	2	5	0	0	332
IC06	0	0	9	25	21	65	0	72	0	5	12	213	0	0	11	3	436
IC07	0	0	0	0	0	10	0	0	0	0	0	0	0	0	0	0	10
OP01	0	0	0	0	0	0	0	0	0	0	0	0	0	0	0	0	0
OP02	0	0	0	0	0	0	0	0	0	0	0	0	0	0	0	0	0
OP03	0	0	0	0	0	0	0	0	0	0	0	0	0	0	0	0	0
OP04	0	0	0	0	0	0	0	0	0	0	0	0	0	0	0	0	0
OP05	0	0	0	0	0	0	0	0	0	0	0	0	0	0	0	0	0
OP06	0	0	0	0	0	0	0	0	0	0	0	0	0	0	0	0	0
OP07	0	0	0	0	0	0	0	0	0	0	0	0	0	0	0	0	0
OP08	0	0	0	0	0	0	0	0	0	0	0	0	0	0	0	0	0

OP09	0	0	0	0	0	0	0	0	0	0	0	0	0	0	0	0	0
OP10	0	0	1	0	0	0	0	0	0	0	0	0	0	0	0	0	1
OP11	0	4	0	4	0	1	3	0	0	0	0	0	0	15	0	12	39
OP12	0	0	0	0	0	6	0	0	7	0	13	0	0	0	29	16	71
OP13	0	0	0	0	0	0	0	0	0	0	0	0	0	0	0	0	0
OP14	0	0	0	0	0	0	0	0	0	0	0	0	0	0	0	0	0
OP15	5	7	0	0	0	0	0	0	0	0	0	0	153	0	0	0	165
OP16	0	0	0	0	0	0	0	0	0	0	0	0	0	0	0	0	0
OP17	0	0	0	0	0	0	0	0	0	0	0	0	0	0	0	0	0
OP18	0	0	0	0	0	0	0	0	0	0	0	0	0	0	0	0	0
OP19	0	0	35	0	0	0	0	0	0	0	0	0	0	101	0	0	136
OP1S	0	0	0	0	0	0	0	312	0	0	0	0	0	0	0	0	312
OP20	0	31	101	0	0	20	37	0	0	3	25	0	0	41	23	0	281
OP21	0	15	23	0	7	0	263	32	0	0	0	10	0	8	0	0	358
OP22	0	0	0	0	0	265	0	12	21	12	0	0	10	54	34	0	408
OP23	0	0	0	0	0	0	0	0	0	0	0	0	0	0	0	0	0
OP24	0	0	0	0	0	0	0	0	0	26	0	0	0	0	0	0	26
OP25	0	0	0	0	0	0	0	0	0	0	0	0	0	0	0	0	0
OP26	0	0	0	0	0	0	0	0	0	0	3	0	0	0	0	0	3
OP27	0	0	0	0	0	0	0	0	0	0	0	0	0	0	0	0	0
OP28	0	0	0	0	0	0	0	0	0	0	0	0	0	0	4	0	4
OP29	0	0	0	0	0	0	0	0	0	0	0	0	0	0	0	0	0
OP30	0	0	0	0	0	0	0	0	0	0	0	0	0	0	0	0	0
OP31	0	0	0	0	0	0	0	0	0	0	0	0	0	0	0	0	0
OPS1	0	0	22	3	0	0	0	0	0	0	0	0	0	0	0	0	25
ZZ01	0	0	0	0	0	0	74	0	0	0	0	0	0	0	0	0	74
ZZ02	0	0	0	0	0	0	0	0	0	0	0	0	0	0	0	0	0
ZZ1S	0	0	0	0	0	0	0	57	110	0	11	2,835	0	0	11	9	3,033
ZZ2S	0	0	0	0	0	0	0	138	44	0	0	896	0	0	0	0	1,078
ZZS1	0	0	65	0	0	0	0	0	0	0	0	0	0	75	1,665	16	1,821
ZZS2	0	0	26	0	0	0	0	0	0	0	0	0	0	5	504	11	546
NANT	0	0	0	0	0	0	0	0	0	0	0	0	0	0	0	0	0
sum	19,032	19,691	15,977	17,823	18,044	19,899	17,270	18,605	15,451	14,096	16,618	29,345	18,051	18,910	27,784	29,669	316,265

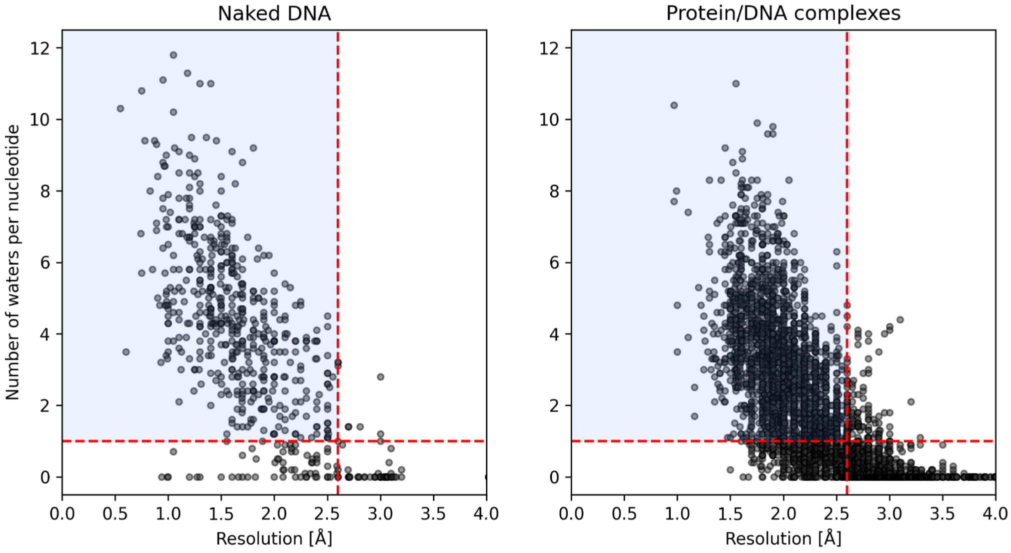

Figure S1. Predicted minor groove hydration in the Z-DNA structure 7jy2 {Harp, 2021 #6859}. Predicted HSs for bases and sugar-phosphate backbone are shown as cyan and yellow spheres, respectively; the corresponding hydration probability densities are shown as cyan and yellow mesh. The HSs forming a spine of hydration along the DNA axis link the O2(C) atoms from opposing strands. The HSs of the N2(G) atoms form water bridges with phosphate OP2 atoms.

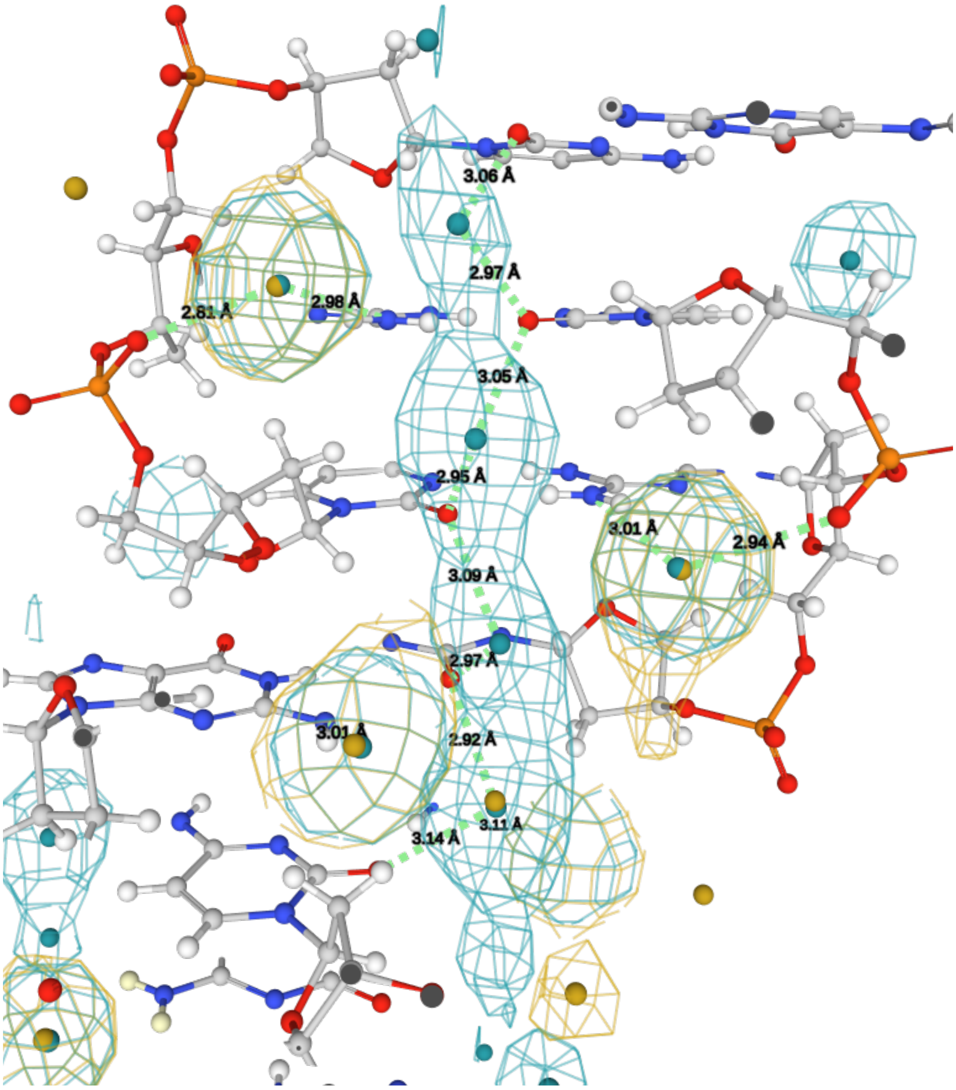

Figure S2. Predicted minor groove hydration in the Z-DNA structure 7jy2 {Harp, 2021 #6859}. Predicted HSs for bases and sugar-phosphate backbone are shown as cyan and yellow spheres, respectively; the corresponding hydration probability densities are shown as cyan and yellow mesh. The HSs forming a spine of hydration along the DNA axis link the O2(C) atoms from opposing strands. The HSs of the N2(G) atoms form water bridges with phosphate OP2 atoms.

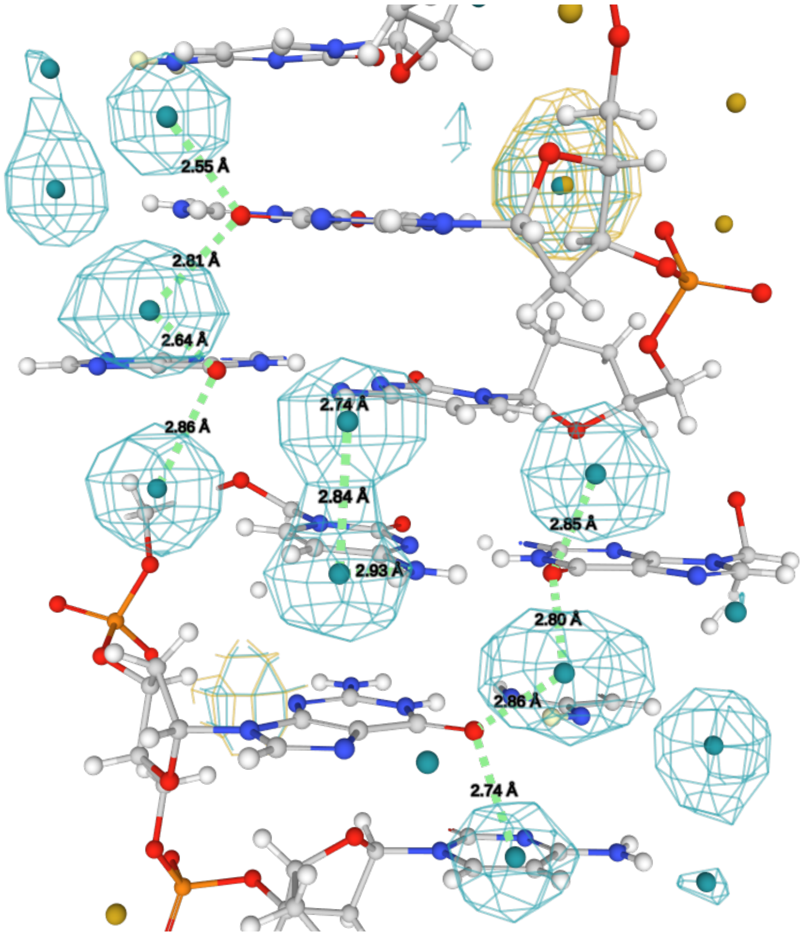

Figure S3. Predicted major groove hydration in the Z-DNA structure 7jy2 {Harp, 2021 #6859}. Predicted HSs for bases and sugar-phosphate backbone are shown as cyan and yellow spheres, respectively; the corresponding hydration probability densities are shown as cyan and yellow mesh. The O6(G) atoms have two HSs lying between the base planes, the one further along the 5' to 3' direction forms an O6(G)...HS...O6(G) bridge with the preceding guanine in the opposing strand. The N4(C) atoms are hydrated by a single HS in plane with the cytosine. The HSs of the cytosines in the opposing strands are aligned vertically along the helical axis and form a double water bridge N4(C)...HS...HS...N4(C).
